# Identification of Vesicle‐Mediated Transport‐Related Genes for Predicting Prognosis, Immunotherapy Response, and Drug Screening in Cervical Cancer

**DOI:** 10.1002/iid3.70052

**Published:** 2024-11-08

**Authors:** Shuai Lou, Hongqing Lv, Lin Zhang

**Affiliations:** ^1^ Department of Gynecology, Affiliated Jinhua Hospital Zhejiang University School of Medicine Jinhua Zhejiang China; ^2^ Department of Gynecology Jinhua Maternal and Child Health Hospital Jinhua Zhejiang China

**Keywords:** cervical cancer, prognosis, tumor immune microenvironment, vesicle‐mediated transport‐related genes

## Abstract

**Background:**

Cervical cancer is one of the most common malignancies among women. Vesicle‐mediated transport mechanisms significantly influence tumor cell behavior through intercellular material exchange. However, prognostic significance in CC patients remains underexplored.

**Research Design and Methods:**

We identified differentially expressed vesicle‐mediated transport‐related genes from TCGA and GeneCards datasets through differential expression analysis. We constructed a prognostic model using Cox regression and LASSO regression, categorized patients into high‐ and low‐risk groups, and validated the model in the GEO data set. A nomogram integrating clinical features and risk scores demonstrated the model's independent prognostic capability. We analyzed tumor immune cell infiltration, immune checkpoints, and predicted immunotherapy responses in the high‐ and low‐risk groups. Finally, we screened potential drugs for targeting CC and conducted drug‐sensitivity analysis.

**Results:**

We successfully established a 10‐gene prognostic model based on VMTRGs. The low‐risk group exhibited favorable prognosis, significant immune cell infiltration, and promising immunotherapy response, whereas the high‐risk group showed higher sensitivity to chemotherapeutic agents such as Docetaxel and Paclitaxel. Potential drugs identified for targeting CC patients included Megestrol acetate, Lenvatinib, Adavosertib, and Barasertib.

**Conclusions:**

The VMTRG‐based prognostic model demonstrates reliable clinical prognostic value and enhances understanding of vesicle‐mediated transport mechanisms in CC.

## Summary

- This study constructed a cervical cancer prognosis model based on vesicle‐mediated transport‐related genes, which demonstrated good predictive performance.
- The study found that patients in the low‐risk group had better prognoses and higher levels of immune cell infiltration, potentially offering advantages for immunotherapy.
- The study also found that patients in the high‐risk group might have higher sensitivity to chemotherapy drugs.

## Graphical abstract

The flow chart provides an overview of the primary design of this study, as illustrated in the figure below.

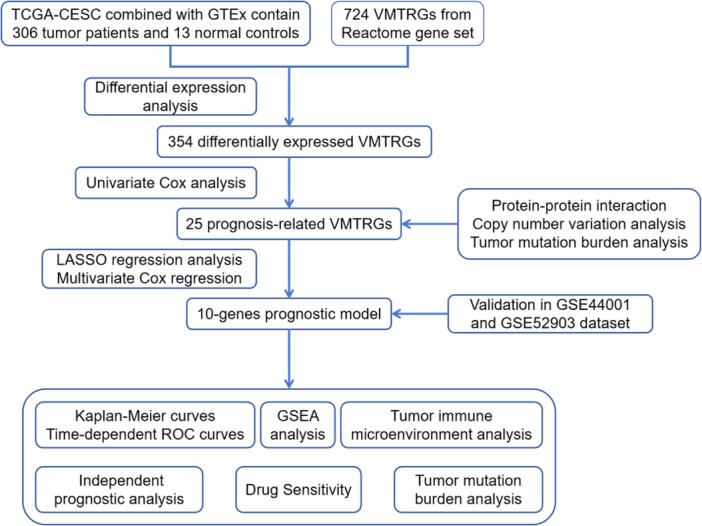

## 1 Introduction

Cervical cancer (CC) is the fourth most common cancer among women in terms of incidence and mortality. According to GLOBOCAN estimates, there were 660,000 new cases and 350,000 deaths worldwide in 2022, imposing a significant healthcare burden [1]. CC is the fourth most common cancer among women in terms of incidence and mortality. According to GLOBOCAN estimates, there were 660,000 new cases and 350,000 deaths worldwide in 2022, imposing a significant healthcare burden [2]. Current CC treatments include surgery (e.g., conization, hysterectomy), radiotherapy, chemotherapy, and targeted therapy, requiring effective combinations based on CC staging and individual differences to achieve optimal therapeutic outcomes [3, 4, 5, 6]. Squamous cell carcinoma is the most common subtype of CC, while adenocarcinoma accounts for approximately 25% of CC cases [7]. PD‐L1 is more frequently expressed by squamous cell carcinoma than by adenocarcinoma. Diffuse PD‐L1 expression in squamous cell carcinoma patients is correlated with poor disease‐free survival and disease‐specific survival compared with marginal PD‐L1 expression, which is associated with a remarkably favorable prognosis. In adenocarcinoma, there is a survival benefit for patients with tumor lacking PD‐L1‐positive tumor‐associated macrophages [8]. However, the prognosis for CC patients remains unsatisfactory, especially for those with metastatic or recurrent disease [9]. A retrospective study of CC patients treated at the Royal Marsden Hospital in the United Kingdom from 2004 to 2014 revealed that 70% of women with recurrent or metastatic CC who received systemic therapy subsequently underwent second‐line treatment. The response rate was poor, with an objective response rate of 13.2%. The median progression‐free survival was 3.2 months, and the median overall survival was 9.3 months [10]. Thus, identifying promising prognostic biomarkers is crucial for aiding clinical decision‐making and improving patient outcomes.

Vesicles transport proteins, lipids, and other macromolecules between cellular compartments or from inside to outside the cell, playing vital roles in cell communication and homeostasis [11, 12]. Molecules carried by extracellular vesicles, such as PD‐L1, have the potential to serve as reliable biomarkers for immunotherapies [13]. Moreover, the cargo transported by exosomes, particularly the range of miRNAs, holds potential as diagnostic biomarkers for numerous cancers, offering valuable targets for early detection and monitoring of the disease, as well as potential interventions [14]. Vesicle transport dysregulation can lead to various diseases, including Alzheimer's [15], cystic fibrosis [16], primary immunodeficiency [17], diabetes [18], and numerous cancers [19, 20, 21, 22]. Increasing evidence suggests that the abnormal expression of vesicle‐mediated transport‐related genes (VMTRGs) plays a crucial role in the development and progression of malignancies. For example, the silencing of the COPB2 gene, involved in Golgi budding and vesicle transport, inhibits colorectal cancer cell proliferation and induces apoptosis via the JNK/c‐Jun signaling pathway [23]. The TMED3 gene in the TMED family promotes the development of malignant melanoma by targeting CDCA8 and regulating the PI3K–AKT signaling pathway [24]. SIRT7 promotes the proliferation and migration of anaplastic thyroid cancer cells by regulating the desuccinylation of KIF23 [25]. Despite these findings, the relationship between VMTRGs and prognosis in CC patients has not been elucidated.

The tumor microenvironment (TME) refers to the complex ecological environment surrounding tumor cells, and targeted therapy strategies specific to the TME have shown promising potential [26]. Immunoregulation within the TME significantly impacts tumor progression, and vesicles play an essential role in tumor immunity. For instance, exosomes derived from gastric cancer cells induce neutrophil autophagy via the HMGB1/TLR4/NF‐κB signaling pathway, promoting tumor activation and remodeling the TME [27]. Extracellular vesicles from melanoma cells increase IFN‐γ secretion, enhancing NKp30 ligand (BAG6, BAT3) expression on their surface, which activates cytotoxic NK cells and increases their tumoricidal activity [28]. Therefore, exploring the impact of VMTRGs on immunity within the TME of CC patients is crucial.

In this study, we obtained CC transcriptome data from accessible public databases and established a VMTRG‐based prognostic risk model. We assessed the model's predictive ability for survival outcomes, immune cell infiltration, and immunotherapy response in CC patients. We further conducted a comprehensive evaluation of the model's clinical applicability. Our comprehensive analysis contributes to a deeper understanding of VMTRG characteristics in CC patients, providing insights for more precise individualized treatment.

## 2 Materials and Methods

### 2.1 Data Collection

Transcriptome data, copy number variation (CNV) data, and corresponding clinical data of CC patients were retrieved from The Cancer Genome Atlas (TCGA) database (https://portal.gdc.cancer.gov) and the Gene Expression Omnibus (GEO) database. Patients lacking survival information were excluded. We collected two GEO cohorts (GSE44001 and GSE52903) as validation sets. Additionally, we obtained transcriptome data from 10 healthy cervical tissues from the GTEx database (https://xenabrowser.net/datapages/). A total of 724 VMTRGs were acquired from the Reactome gene set (Table S1).

### 2.2 Identification and Analysis of Prognostic Differentially Expressed VMTRGs

We combined TCGA and GTEx datasets, totaling 306 CC samples and 13 healthy tissues. Differential expression analysis was conducted using the “limma” package and Wilcoxon test, applying thresholds of false discovery rate (FDR) < 0.05 and |log2 fold‐change (FC)| > 0.585 to identify differentially expressed genes (DEGs). The intersecting DEGs and VMTRGs were considered differentially expressed VMTRGs (DEVMTRGs). Concurrently, univariate Cox regression analysis was performed to screen for prognostic DEVMTRGs (*p* < 0.01). Correlation analysis of prognostic DEVMTRGs was conducted, and a boxplot was drawn to visualize the differences between tumor and healthy tissues. The STRING database (https://string-db.org/) was used to construct a protein–protein interaction (PPI) network. CNV analysis was performed to determine the chromosomal distribution of these genes. The “maftools” package was used to generate a TMB waterfall plot of prognostic DEVMTRGs.

### 2.3 Creation, Evaluation, and Validation of Prognostic Risk Signature

Due to the impracticality of using a large number of genes in clinical testing, we performed LASSO Cox regression analysis using the “glmnet” package to prevent overfitting by introducing a penalty parameter lambda during 10‐fold cross‐validation. Subsequently, multivariate Cox regression analysis was conducted based on the genes selected by LASSO to construct a RiskScore for CC prognosis assessment, calculated as follows:

$$\text{Riskscore}\;=\sum_{i=1}^{n}\beta_{i}*\text{Exp}\;i,$$

where $\beta_{i}$ represents the gene coefficient and Expi represents the relative expression level of the gene.

The RiskScore was calculated for TCGA, GSE44001, and GSE52903 cohorts. Based on the median RiskScore, samples were divided into high‐ and low‐risk groups. Kaplan–Meier (K–M) survival curves were plotted to assess prognostic differences between the groups. The “timeROC” package was used to plot the receiver operating characteristic (ROC) curve, evaluating the RiskScore's performance in predicting CC prognosis. Additionally, scatter plots of survival time and risk scores for each patient were generated.

### 2.4 Functional Enrichment Analysis

Gene Set Enrichment Analysis (GSEA) 4.3.0 software was used with Kyoto Encyclopedia of Genes and Genomes (KEGG) datasets to plot enrichment pathways for high‐ and low‐RiskScore groups. Differentially expressed genes between high‐ and low‐RiskScore groups (FDR < 0.05, |log2 FC | > 1) were subjected to Gene Ontology (GO) and KEGG pathway enrichment analyses.

### 2.5 Application and Independent Prognostic Evaluation of the Prognostic Model

Clinical characteristic correlation analysis was conducted on TCGA CC patients to understand the interaction between RiskScore and clinical features. Survival analysis of model feature genes was performed, and K–M curves were plotted. Univariate and multivariate Cox regression analyses were carried out, combining clinical characteristics and RiskScore to determine if RiskScore could serve as an independent prognostic indicator. The “rms” package was used to plot a nomogram incorporating RiskScore and other clinical features. Calibration curves and decision curve analysis (DCA) curves were used to evaluate the predictive performance of the nomogram.

### 2.6 Immune Microenvironment Analysis and Immunotherapy Response Prediction

The ssGSEA algorithm was used to calculate immune infiltration scores for 29 immune cell types or pathways. The “estimate” package was employed to estimate and compare the immune score, stromal score, ESTIMATE score, and tumor purity between high‐ and low‐RiskScore groups. To ensure robustness, the CIBERSORT algorithm was also utilized. Expression differences of immune‐related genes between high‐ and low‐RiskScore groups were analyzed. The immune profile score (IPS) for each sample was obtained from The Cancer Immunome Atlas (TCIA) database.

### 2.7 Tumor Mutation Burden and Drug Sensitivity Assessment

To differentiate the mutation profiles of CC patients between high‐ and low‐RiskScore groups, MAF files from the TCGA database were obtained and waterfall plots were generated using the “maftools” package. To investigate the clinical performance of chemotherapy drugs in CC patients, the “pRRophetic” package was used to calculate the half‐maximal inhibitory concentration (IC_50_) values of common drugs. The CellMiner database was used to reveal the correlation between model feature genes and anticancer drug sensitivity. Spearman correlation analysis was conducted to determine the relationship between the expression of risk genes and drug sensitivity.

### 2.8 Statistical Analysis

All analyses were conducted using Perl (version 5.32.1), R (version 4.1.3), and related packages. The Wilcoxon test was used to determine statistical differences between groups. K–M survival curve analysis employed the log‐rank test to determine *p* values between groups. ROC curves were plotted using the “timeROC” package. Data visualization was primarily performed using the “ggplot2” package. *p* values were summarized as follows: ****p < 0.0001; ***0.0001 < *p* < 0.001; **0.001 < *p* < 0.01; *0.01 < *p* < 0.05; ^ns^ *p* > 0.05.

## 3 Results

### 3.1 Transcriptional and Genetic Alterations of VMTRGs in CC Patients

**Figure 1:**
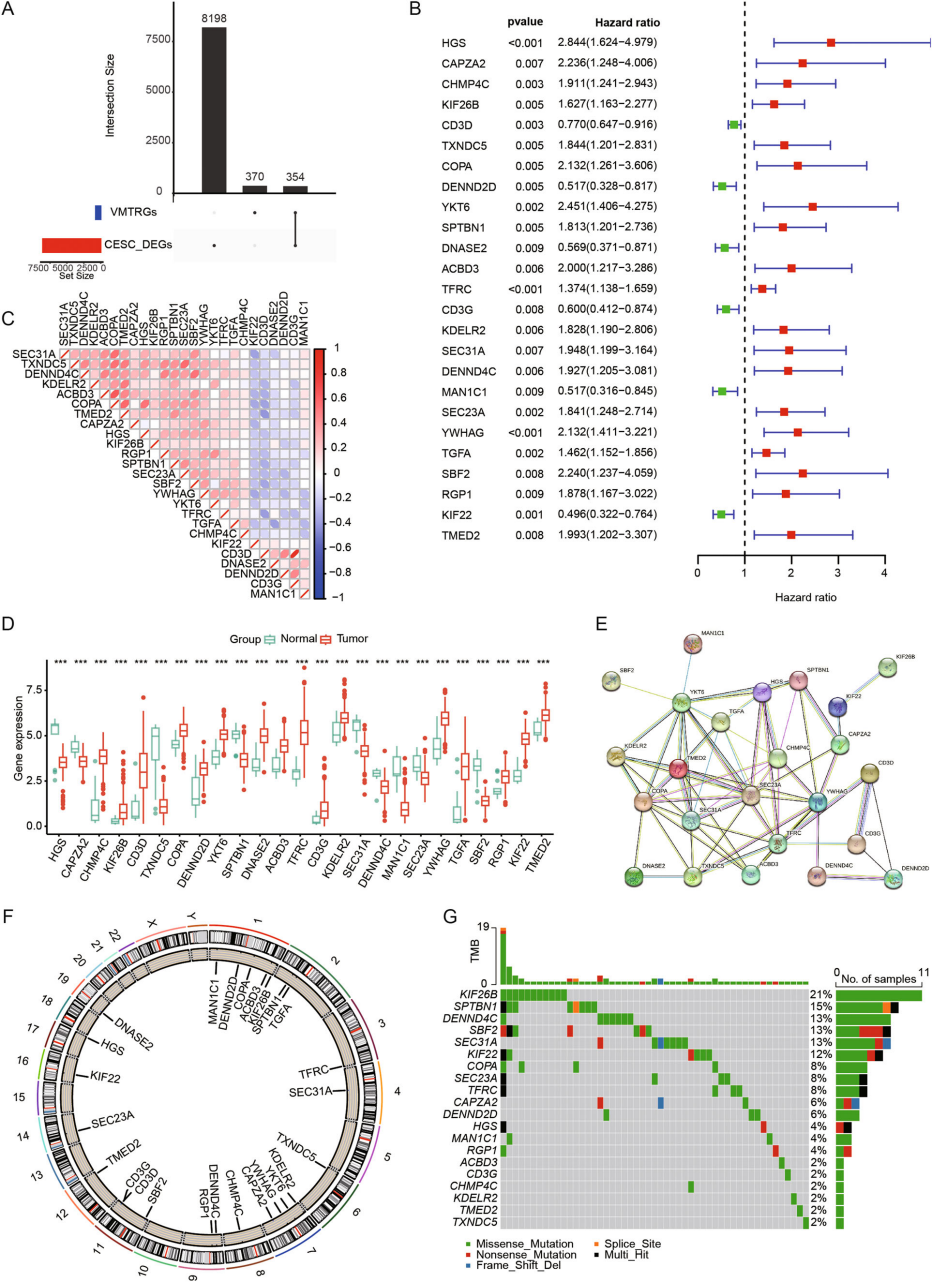
Genomic and transcriptomic landscape of VMTRGs. (A) Intersection analysis of DEGs and VMTRGs. (B) Forest plot of univariate Cox analysis for prognostic genes. (C) Correlation heatmap of vesicle‐mediated transport‐related prognostic genes. (D) Box plot of differential expression of vesicle‐mediated transport‐related prognostic genes between tumor and normal tissues. (E) PPI network of vesicle‐mediated transport‐related prognostic genes. (F) CNV analysis of vesicle‐mediated transport‐related prognostic genes. (G) Mutation waterfall plot of vesicle‐mediated transport‐related prognostic genes.

Through differential analysis of tumor and normal samples from TCGA combined with GTEx, we identified 8552 differentially expressed genes (DEGs). By intersecting these DEGs with VMTRGs, we obtained 354 DE‐VMTRGs in CC patients (Figure 1A and Table S2). Univariate Cox analysis identified 25 genes potentially associated with CC prognosis (Figure 1B). Correlation heatmap analysis revealed strong correlations among these 25 genes (Figure 1C and Table S3). We compared their expression patterns in normal and tumor tissues within the TCGA‐GTEx cohort. Among these, 16 genes (CHMP4C, KIF26B, CD3D, COPA, DENND2D, YKT6, DNASE2, ACBD3, TFRC, CD3G, KDELR2, YWHAG, TGFA, RGP1, KIF22, and TMED2) were significantly upregulated in CC, while 9 genes (HGS, CAPZA2, TXNDC5, SPTBN1, SEC. 31 A, DENND4C, MAN1C1, SEC. 23 A, and SBF2) were significantly downregulated (Figure 1D). Using the STRING database, we constructed a PPI network among these 25 genes, demonstrating close connections (Figure 1E). CNV analysis indicated CNVs on multiple chromosomes, particularly on chromosomes 127 and 911 (Figure 1F). Mutation analysis in the TCGA CC cohort revealed mutations in these genes across 52 samples, with the highest mutation frequency observed in KIF26B, followed by SPTBN1, DENND4C, SBF2, and SEC. 31 A (Figure 1G).

### 3.2 Construction and Validation of a Prognostic Model for Vesicle‐Mediated Transport in CC

**Figure 2:**
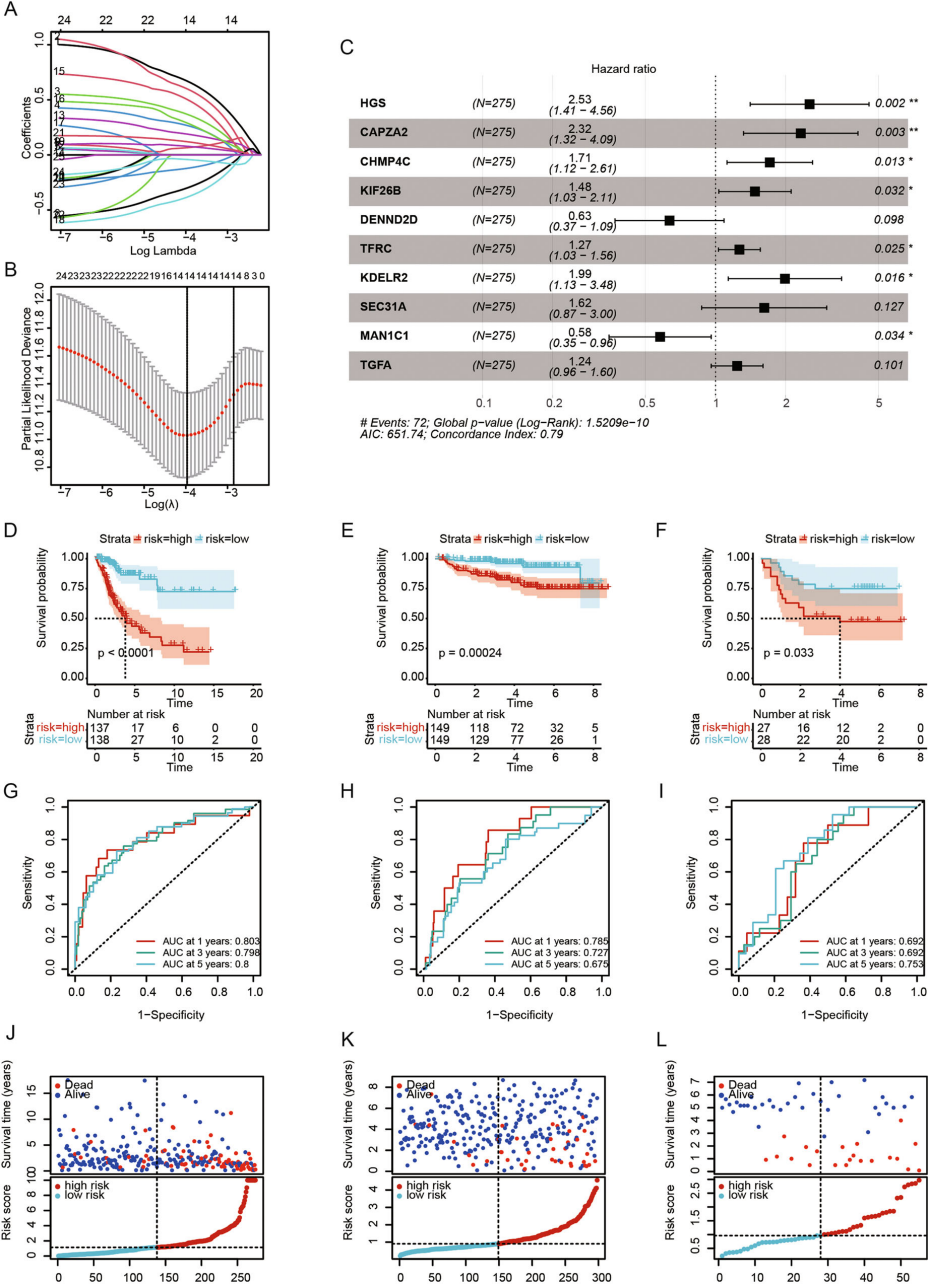
Construction and validation of a prognostic model for vesicle‐mediated transport in CC. (A) Coefficient distribution plot for log(*λ*) sequence in the LASSO model. (B) LASSO coefficient spectrum from LASSO Cox analysis. (C) Forest plot of multivariate Cox regression analysis. (D) Kaplan–Meier survival curve of overall survival in high‐ and low‐risk groups in the TCGA training set. (E) Kaplan–Meier survival curve in the GSE44001 validation set. (F) Kaplan–Meier survival curve in the GSE52903 validation set. (G) AUC curves for the TCGA training set at 1, 3, and 5 years. (H) AUC curves for the GSE44001 validation set at 1, 3, and 5 years. (I) AUC curves for the GSE52903 validation set at 1, 3, and 5 years. (J–L) Distribution plots of risk scores, survival time, and survival status in the TCGA and GEO datasets.

To identify the optimal candidate genes, LASSO Cox regression analysis was used to screen 14 genes from the previous 25 (Figure 2A,B and Table S4). Multivariate Cox regression analysis of these 14 genes yielded a 10‐ge prognostic model:

Riskscore=0.9299×HGS+0.8427×CAPZA2+0.5364×CHMP4C+0.3901×KIF26B−0.4542×DENND2D+0.2361×TFRC+0.6859×KDELR2+0.4815×SEC.31A−0.5474×MAN1C1+0.2142×TGFA.

Patients were divided into high‐ and low‐risk groups based on the median RiskScore. To evaluate the robustness of the risk model based on the TCGA training set, external validation was performed using GEO validation sets. K–M survival analysis indicated worse prognostic outcomes for the high‐risk group in both the TCGA training set and GEO validation sets (Figure 2D–F). ROC curves were used to assess the predictive performance of the prognostic model, with AUC values of 0.803, 0.798, and 0.8 for 1, 3, and 5 years in the TCGA cohort (Figure 2G), and 0.785, 0.727, and 0.675 for the GSE44001 validation set (Figure 2H), and 0.692, 0.692, and 0.753 for the GSE52903 validation set (Figure 2I). The distribution of risk scores, survival time, and survival status showed consistent trends in both the TCGA and GEO datasets (Figure 2J–L). Our results indicate that the constructed prognostic model has significant prognostic predictive value.

### 3.3 Significant Enrichment Pathway Analysis between High‐ and Low‐Risk Groups

**Figure 3:**
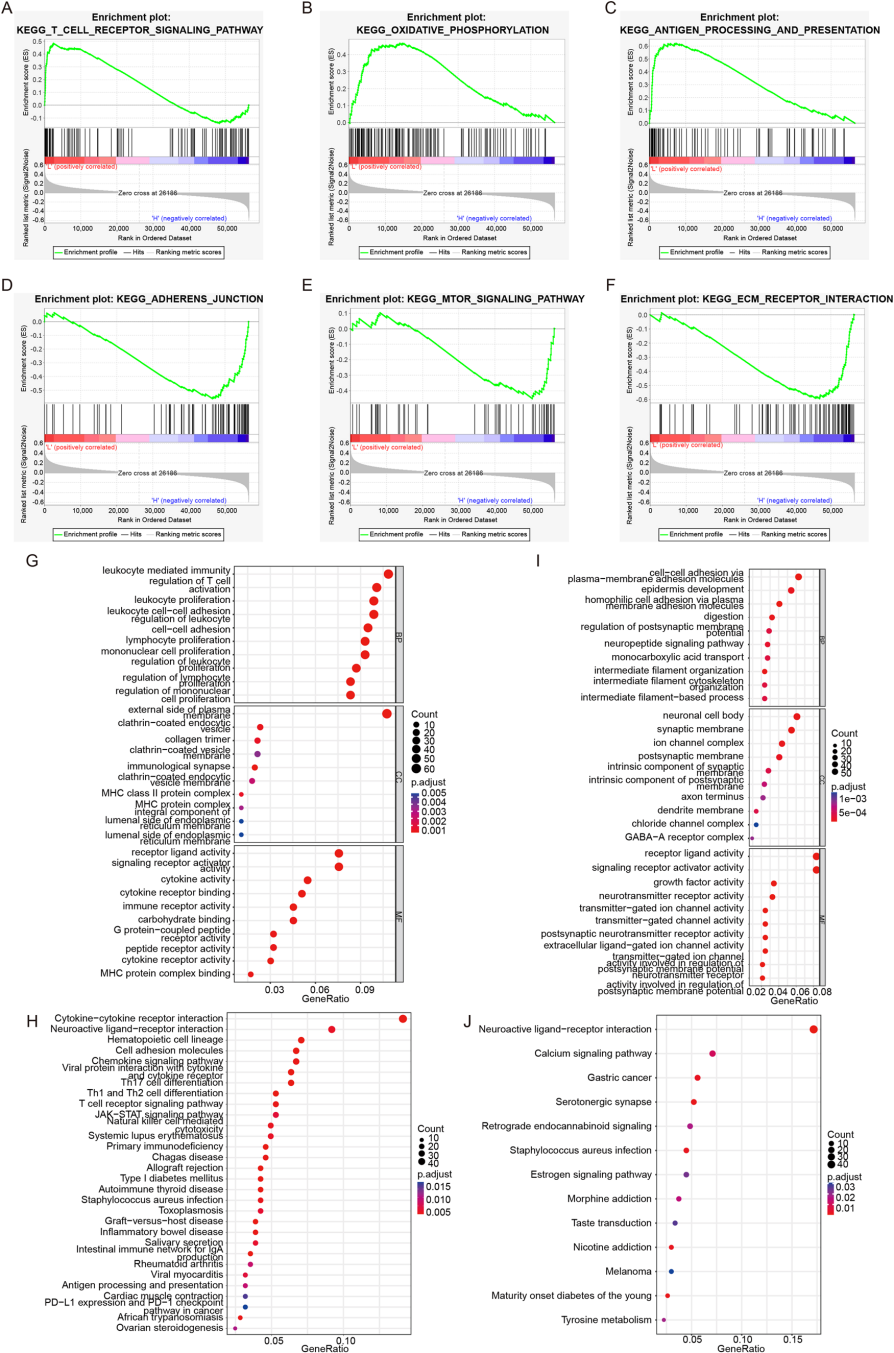
Significant enrichment pathways between high‐ and low‐risk groups. (A–C) GSEA analysis of low‐risk groups. (D–F) GSEA analysis of high‐risk groups. (G) GO enrichment analyses of low‐risk groups: BP, biological processes; CC, cellular components; MF, molecular functions. (H) KEGG analysis of low‐risk groups. (I) GO enrichment analyses of high‐risk groups. (J) KEGG analysis of high‐risk groups.

To decode specific biological characteristics between high‐ and low‐risk groups, we used GSEA software with gene sets from KEGG. The low‐risk group was significantly enriched in pathways such as T CELL RECEPTOR, OXIDATIVE PHOSPHORYLATION, and ANTIGEN PROCESSING AND PRESENTATION (Figure 3A–C), while the high‐risk group was enriched in pathways such as ADHERENS JUNCTION, MTOR SIGNALING PATHWAY, and ECM RECEPTOR INTERACTION (Figure 3D–F). Differential analysis (|logFC| > 1, adj.*p* < 0.05) identified 1273 DEGs between the high‐ and low‐risk groups. GO and KEGG enrichment analyses of upregulated genes in the low‐risk group revealed associations with leukocyte‐mediated immunity and enrichment in cytokine–cytokine receptor interaction (Figure 3G,H). The high‐risk group was associated with cell–cell adhesion via plasma membrane adhesion molecules and enriched in neuroactive ligand–receptor interaction (Figure 3I,J).

### 3.4 Independent Prognostic Analysis of the Risk Model

**Figure 4:**
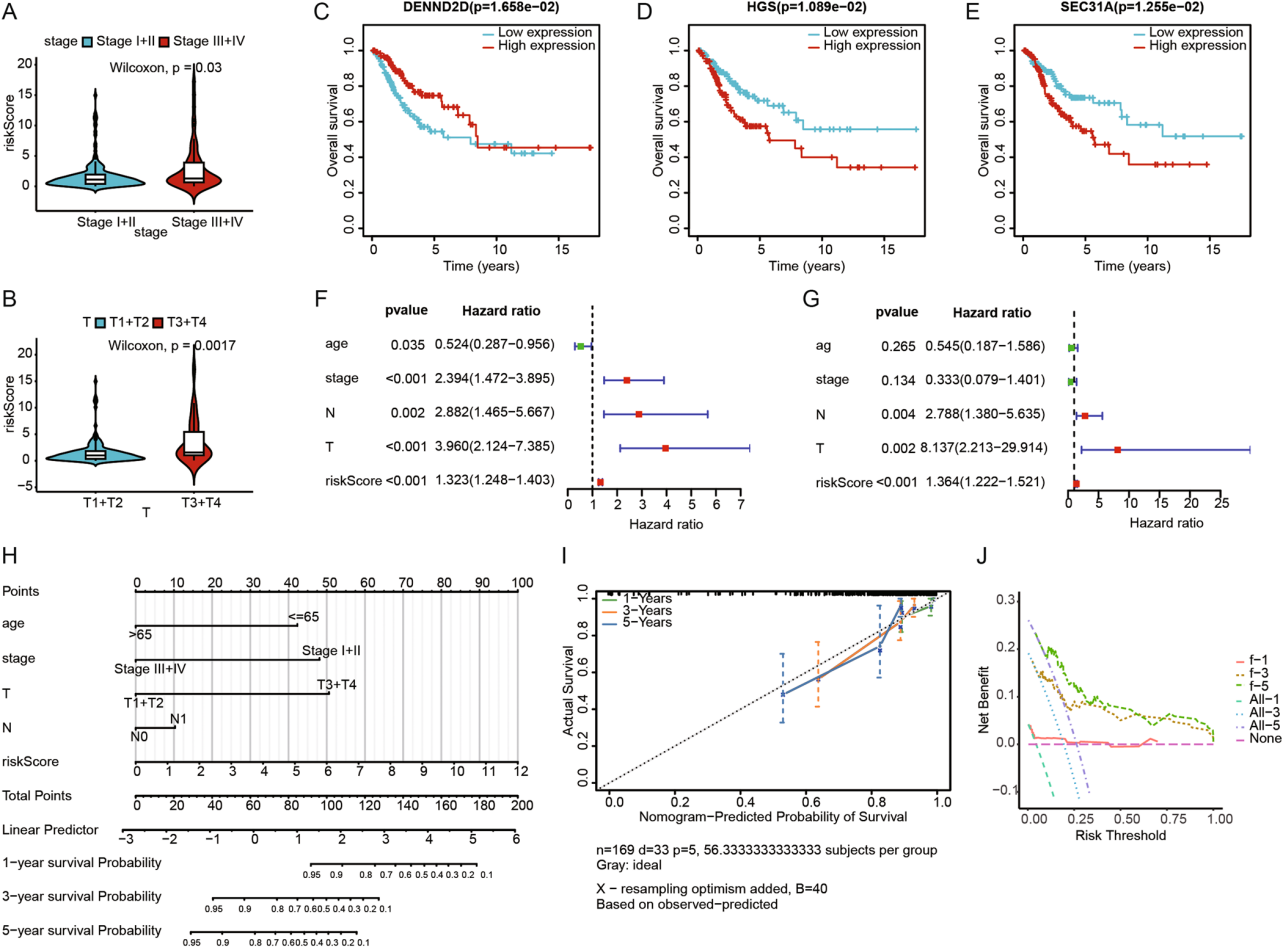
Independent prognostic analysis of the model. (A) The difference in risk score by stage of CESC. (B) The difference in risk score by T stage of CESC. (C) Kaplan–Meier curves for DENND2D in the TCGA‐CESC cohort. (D) Kaplan–Meier curves for HGS in the TCGA‐CESC cohort. (E) Kaplan–Meier curves for SEC. 31A in the TCGA‐CESC cohort. (F, G) Univariate and multivariate Cox regression analyses. (H) The predictive nomogram. (I) Calibration curves for 1, 3, and 5 years. (J) DCA curves for 1‐, 3‐, and 5‐year OS in CESC.

We explored the association between clinical characteristics and risk scores, finding statistically significant differences in risk scores for stage and T stage (Figure 4A,B). Survival analysis of the 10 characteristic genes identified DENND2D, HGS, and SEC. 31 A as having survival differences in the TCGA‐CESC cohort (Figure 4C–E). Univariate and multivariate Cox regression analyses were performed to evaluate the independent prognostic performance of the model, revealing that risk scores were significantly associated with prognosis (Figure 4F), and multivariate Cox regression analysis confirmed risk scores as an independent prognostic factor (Figure 4G). Based on the TCGA cohort, we constructed a nomogram combining clinical characteristics and risk scores to predict the prognosis of CESC patients (Figure 4H). The calibration curves for 1, 3, and 5 years showed high concordance between the predicted and actual survival rates (Figure 4I), and the DCA curves indicated strong predictive probabilities for 1‐, 3‐, and 5‐year overall survival (Figure 4J).

### 3.5 Analysis of Immune Landscape and Immunotherapy Response in the Prognostic Model

**Figure 5:**
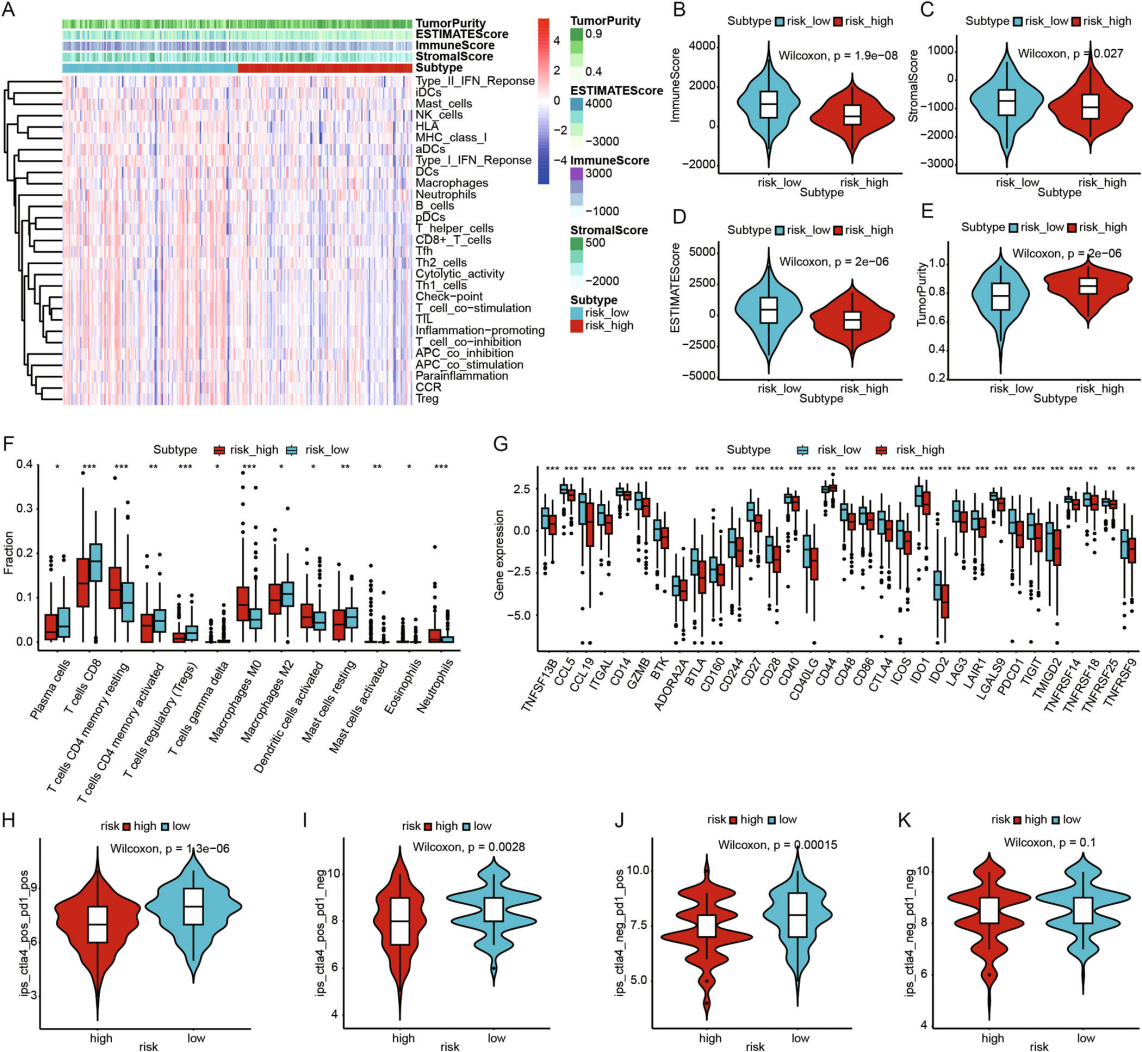
Analysis of the immune landscape and immune therapy response of the prognostic model. (A) Heatmap of ssGSEA immune infiltration differences between high‐ and low‐risk groups. (B) Comparison of the immune score between high‐ and low‐risk groups. (C) Comparison of the stroma score between high‐ and low‐risk groups. (D) Comparison of the ESTIMATE score between high‐ and low‐risk groups. (E) Comparison of the tumor purity between high‐ and low‐risk groups. (F) Box plot showing the differences in immune cell infiltration between low‐ and high‐risk groups based on CIBERSORT. (G) Differential expression of immune checkpoint molecules between low‐ and high‐risk groups. (H–K) IPS scores in high‐ and low‐risk groups.

We used ssGSEA to explore differences in immune‐related functions and immune cell infiltration between high‐ and low‐risk groups. As shown in the figure, the low‐risk group exhibited higher levels of immune‐related functions and immune cell infiltration compared to the high‐risk group (Figure 5A). The ESTIMATE algorithm assessment indicated that the low‐risk group had significantly higher Immune Score, Stroma Score, and ESTIMATE Score, and significantly lower Tumor Purity compared to the high‐risk group (Figure 5B–E). CIBERSORT algorithm results showed that the infiltration levels of plasma cells, CD8 T cells, activated CD4 memory T cells, regulatory T cells (Tregs), gamma delta T cells, M2 macrophages, and resting mast cells were significantly higher in the low‐risk group. Conversely, the high‐risk group had significantly higher infiltration levels of resting CD4 memory T cells, M0 macrophages, activated dendritic cells, and neutrophils (Figure 5F).

Next, we assessed the differential expression of immune‐related genes between the high‐ and low‐risk groups. The results revealed that the majority of immune‐related genes were expressed at higher levels in the low‐risk group (Figure 5G). To further evaluate the immune therapy response in CESC patients, we calculated IPS scores. The findings showed that the low‐risk group had higher IPS scores, suggesting that patients in this group might be more sensitive to immune therapy and more likely to benefit from it (Figure 5H–K).

### 3.6 Tumor Mutation Burden Analysis and Drug Sensitivity Analysis

**Figure 6:**
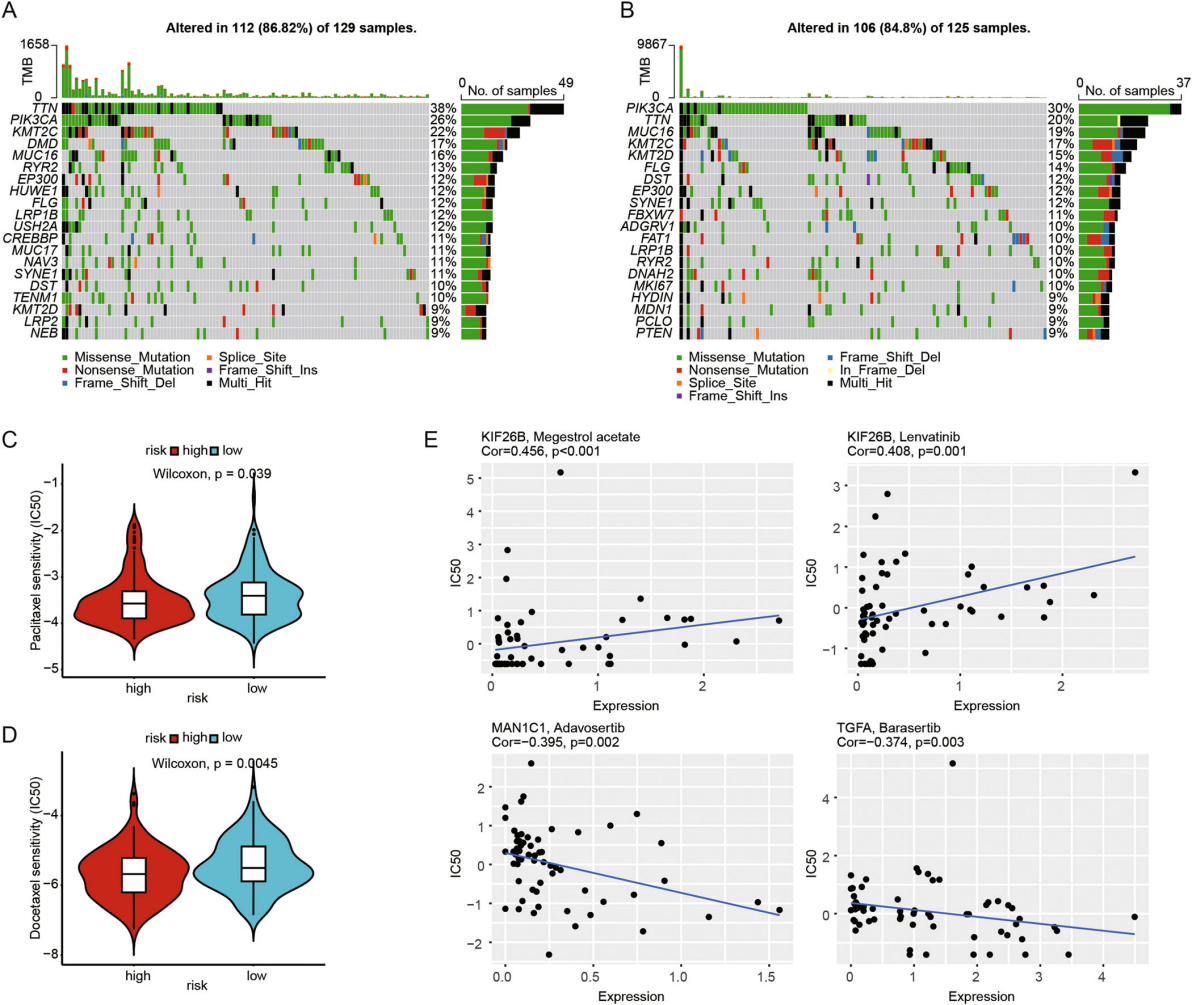
Tumor mutation burden analysis and drug sensitivity analysis. (A) Waterfall plot showing the mutation distribution of the top 20 most frequently mutated genes in the high‐risk group. (B) Waterfall plot showing the mutation distribution of the top 20 most frequently mutated genes in the low‐risk group. (C) Violin plot of Paclitaxel IC_50_ values between high‐ and low‐risk groups. (D) Violin plot of Docetaxel IC_50_ values between high‐ and low‐risk groups. (E) Correlation between model feature gene expression levels and drug sensitivity predicted by the CellMiner database.

To investigate the relationship between somatic mutations and risk scores, we conducted a TMB analysis. The results showed a lower mutation probability in the high‐risk group. Compared to the high‐risk group, patients in the low‐risk group appeared to have a higher trend of TTN mutations, at 38% versus 20%, respectively, while the high‐risk group had a higher trend of PIK3CA mutations, at 30% versus 26% (Figure 6A,B). We used the pRRophetic algorithm to analyze the IC_50_ values of two common chemotherapy drugs (Docetaxel and Paclitaxel) in treating CESC patients. The results indicated that the high‐risk group was more sensitive to Docetaxel and Paclitaxel compared to the low‐risk group (Figure 6C,D). Additionally, we conducted a Sperman correlation analysis to assess the impact of model feature genes on drug sensitivity using the CellMiner database. The results indicated a positive correlation between KIF26B expression and Megestrol acetate, as well as Lenvatinib, and a negative correlation between MAN1C1 and Adavosertib, and TGFA and Barasertib (Figure 6E). These findings suggest that our prognostic model may have a strong correlation with the sensitivity to these drugs.

## 4 Discussion

With the widespread adoption of CC screening and HPV vaccines, significant achievements have been made in CC [29]. NHS England first aimed to tackle the burden of the disease by introducing a national cervical screening program in 1988, which has since seen a significant reduction in over a third of cases in England. CC screening is available from the age of 25, as the disease is rare among younger individuals [30]. However, for patients with CC, particularly those with recurrent or metastatic CC, overall survival (OS) rates remain poor [31]. In recent years, vesicle‐mediated transport has gained increasing attention for its role in tumorigenesis, metastasis, and tumor immunotherapy [32, 33]. For instance, Macitentan, an FDA‐approved oral drug, enhances antitumor immune responses by inhibiting the secretion of PD‐L1 from tumor‐derived extracellular vesicles [34]. Currently, VMTRGs has demonstrated precise prognostic predictions in lung adenocarcinoma and hepatocellular carcinoma [35, 36], but its prognostic significance in CC patients needs comprehensive elucidation. This study utilized the TCGA data set to perform a series of related analyses on VMTRGs, successfully selecting 10 optimal VMTRGs to construct a prognostic risk model. The model showed a significant correlation with the OS of CC patients and was validated using an external validation set. The model demonstrated potential value in predicting the survival, immune infiltration, and immune therapy response of CC patients.

We performed univariate Cox analysis to identify genes significantly associated with prognosis in CESC patients (*p* < 0.01). To minimize model complexity and prevent overfitting, we utilized LASSO regression analysis with 10‐fold cross‐validation, which enhances the model's generalizability and robustness. Ultimately, we applied stepwise multivariate Cox regression analysis to ascertain the optimal model composition and the prognostic relevance of these genes. Hepatocyte growth factor‐regulated tyrosine kinase substrate (HGS) is involved in cytokine and growth factor‐mediated intracellular signaling. Sun et al. found that high levels of HGS expression were associated with poor prognosis in colorectal cancer patients, suggesting that HGS might be a potential therapeutic target for colorectal cancer [37]. Consistent with our findings, HGS is a poor prognostic factor in CESC patients (Figure 1B), with higher HGS expression associated with poorer prognosis (Figure 4D). The CAPZA2 gene encodes a member of the F‐actin capping protein subunit family. Kwon et al. found that CAPZA2 knockdown reduced the proliferation of gastric adenocarcinoma cells by increasing apoptosis and inhibiting cell cycle progression [38]. CHMP4C, a chromatin‐modifying protein, plays a crucial role in many processes as extracellular vesicles, including tumorigenesis and progression [39, 40]. Lin et al. found that CHMP4C is highly expressed in CC cells and may promote malignant progression through epithelial–mesenchymal transition [41]. Carlton et al. discovered that silencing CHMP4C could enhance the sensitivity of lung cancer cells to radiation by delaying the S phase of the cell cycle [42]. KIF26B, a member of the KIF family, consists of 2108 amino acids [43]. Recent studies have found that KIF26B is associated with the occurrence, progression, and metastasis of various solid tumors, such as hepatocellular carcinoma [44], breast cancer [45], and gastric cancer [46]. Chen et al. found that inhibiting KIF26B can suppress non‐small cell lung cancer by affecting the AKT/GSK‐3β/β‐catenin pathway, suggesting that KIF26B might be a potential target [47]. DENND2D, located on chromosome 1p13.3, encodes a 53‐kDa protein and is a member of the DENND2 family [48, 49]. Previous studies have shown that DENND2D acts as a tumor suppressor gene in various cancers, including hepatocellular carcinoma [50], non‐small cell lung cancer [51], and esophageal squamous cell carcinoma [52]. Additionally, Sakha et al. found that exosome‐derived miR‐1246 promotes the migration and invasion of oral squamous cell carcinoma by directly targeting DENND2D [53]. This is consistent with our findings, where DENND2D also acts as a tumor suppressor gene (Figure 4C) and is a protective factor for the prognosis of CESC patients (Figure 2C). TFRC encodes transferrin receptor protein 1, the main cellular iron transporter [54]. Lu et al. discovered that MYCN increases iron uptake by mediating TFRC upregulation and promotes reprogramming of cellular iron metabolism [55]. KDELR2 is a key receptor for transport from the Golgi to the endoplasmic reticulum [56]. Recent studies have shown that KDELR2 is highly expressed in glioblastoma, mediating the phosphorylation level of mTOR, thereby promoting glioblastoma proliferation [57]. SEC. 31A is a component of the outer layer of the coat protein complex II (COPII) [58]. Cheng et al. found that miR‐376a targets and regulates SEC. 31A expression, promoting migration, invasion, glycolysis, and apoptosis in non‐small cell lung cancer [59]. MAN1C1 plays a crucial role in glycosylation, particularly in the modification of proteins through the endoplasmic reticulum and Golgi apparatus [60]. Wei et al. demonstrated that the loss of MAN1C1 promotes CD133 glycosylation in intrahepatic cholangiocarcinoma cells, enhancing CD133‐FIP200 interaction and promoting tumorigenesis in intrahepatic cholangiocarcinoma [61]. TGFA encodes a growth factor that is a member of the epidermal growth factor family, regulating autocrine in breast cancer cells [62]. It has been reported that circTAF4B promotes TGFA expression by sponging miR‐1298‐5p, thereby promoting proliferation and epithelial–mesenchymal transition in bladder cancer cells [63].

Through GSEA enrichment analysis, we found that the low‐risk group was mainly enriched in pathways such as T cell receptor (TCR) signaling pathway, oxidative phosphorylation, and antigen processing and presentation. Yuan et al. found that LDLR interacts with the TCR on the plasma membrane of CD8 T cells, promoting TCR signaling and CD8 T cell effector functions [64]. Other studies have found that gut microbiota metabolite butyrate upregulates PD‐1/CD28 expression in CD8 T cells and regulates the TCR signaling pathway, promoting CD8 T cell antitumor immune response [65]. This is consistent with our findings, where we observed higher immune infiltration levels and better prognosis in the low‐risk group. Additionally, the high‐risk group was mainly enriched in pathways such as adherens junction, mTOR signaling pathway, and ECM receptor interaction. Li et al. found that TRIM28 promotes CC cell growth by activating the mTOR signaling pathway [66]. Liu et al.'s research showed that ECT2 is highly expressed in CC and inhibits cancer cell apoptosis by promoting the activation of the AKT/mTOR pathway [67]. Therefore, we speculate that the active mTOR signaling pathway in the high‐risk group leads to poorer prognosis. Although the exact mechanisms of these genes need further exploration, these findings suggest that the identified feature genes can predict the prognosis of CC patients and may serve as promising therapeutic targets.

The tumor immune microenvironment (TIME) is a complex ecosystem surrounding tumor tissues, comprising various immune cells, mechanical cells, blood vessels, and extracellular matrix, playing a crucial role in tumorigenesis, development, metastasis, and treatment [68, 69, 70]. Myeloid‐derived suppressor cells, T regulatory (Treg) cells, and tumor‐associated macrophages constitute immunosuppressive cells present within the TME, which release reactive oxygen species (ROS) amongst other factors, effectively inhibiting natural killer (NK) cell response (22313874). Higher levels of fibroblasts secrete more metalloproteinases, resulting in further shedding of ligands that could link to NK cells. Fibroblasts even have a more direct impact on NK cells by preventing cytokine‐induced activating receptor upregulation [71]. Our study found that the low‐risk group had significantly higher levels of immune cell infiltration, immune scores, and stromal scores compared to the high‐risk group. Previous studies have also shown that high immune cell infiltration is favorable for immunotherapy in tumors [72, 73, 74]. Immune infiltration analysis revealed higher levels of plasma cells, CD8 T cells, activated memory CD4 T cells, regulatory T cells (Treg), M2 macrophages, and resting mast cells in the low‐risk group. Conversely, the high‐risk group showed significantly increased levels of resting memory CD4 T cells, M0 macrophages, activated dendritic cells, and neutrophils. Higher infiltration levels of CD8 and CD4 T cells indicate better prognosis in tumor patients [75]. A higher proportion of resting mast cells has also been associated with longer OS [76], consistent with our conclusion of better prognosis in the low‐risk group. Additionally, we found that the majority of immune checkpoints (including PD‐1, CTLA4, LAG3, TIGIT, etc.) were highly expressed in the low‐risk group, indicating that patients in this group might be more suitable for immune checkpoint inhibitor therapy. Furthermore, the low‐risk group exhibited higher IPS scores, suggesting they might achieve better outcomes from immunotherapy.

During the process of screening feature genes and risk grouping, we also analyzed and identified potential anticancer drugs. We found that two taxane chemotherapy drugs, Docetaxel and Paclitaxel, showed higher drug sensitivity in the high‐risk group, implying that high‐risk patients might achieve better therapeutic outcomes from chemotherapy. Based on the prediction of these feature genes, we identified potential drugs for treating CC patients, such as Megestrol acetate, Lenvatinib, Adavosertib, and Barasertib, aiming to provide insights for CC treatment. Previous studies have shown that Megestrol acetate, a synthetic progestogen, is used in patients with advanced endometrial cancer [77]. Lenvatinib, a multi‐target tyrosine kinase inhibitor, reduces the proliferation of CC cell lines when used in combination with PD‐1/PD‐L1 inhibitors [78]. Adavosertib, a WEE1 kinase inhibitor, has been shown in clinical trials to be effective in treating CC when combined with chemoradiotherapy [79]. Barasertib, an Aurora B kinase inhibitor, has been proven to block the expression of HPV 16E6 and BCL‐2 and increase p53 expression, thereby enhancing cisplatin sensitivity in CC cell line SiHa [80]. Therefore, our research results are expected to provide clues for the personalized treatment of CC patients and insights for future in‐depth drug development.

In conclusion, our study constructed a 10‐gene risk prognostic model that can predict the prognosis of CC patients and can serve as an independent prognostic factor. Based on this model, we found that patients in the low‐risk group had higher levels of immune infiltration and might be more suitable for immunotherapy. Finally, we predicted corresponding chemotherapy and targeted drugs, identifying potential CC treatment drugs. However, our study has certain limitations. First, we used comprehensive bioinformatics analysis of existing public data. As a retrospective study, a larger sample size and prospective cohorts are needed to further validate the model's accuracy. Second, the potential regulatory mechanisms of the 10 feature genes in the occurrence and development of CC still require more exploration.

## 5 Conclusion

We developed a prognostic model based on VMTRGs, which has excellent predictive ability for the prognosis of CC patients and has been identified as an independent prognostic factor. The risk scores of this model are highly correlated with the levels of immune infiltration. Overall, our study provides supportive evidence for vesicle‐mediated transport‐related mechanisms in CC patients.

## Author Contributions

Shuai Lou, Hongqing Lv, and Lin Zhang contributed to the study design. Shuai Lou conducted the literature search. Shuai Lou, Hongqing Lv, and Lin Zhang acquired the data. Shuai Lou and Hongqing Lv wrote the article. Lin Zhang performed data analysis. Shuai Lou drafted. Hongqing Lv and Lin Zhang revised the article and gave the final approval of the version to be submitted. All authors read and approved the final manuscript.

## Ethics Statement

The authors have nothing to report.

## Conflicts of Interest

The authors declare no conflicts of interest.

## Supporting information

Supplementary Table S1: Vesicle‐mediated transport‐related genes.

Supplementary Table S2: 354 intersecting genes.

Supplementary Table S3: Results of Univariate Cox analysis.

Supplementary Table S4: Results of LASSO regression analysis. lasso, least absolute shrinkage and selection operator.

## Data Availability

The data and materials in the current study are available from the corresponding author on reasonable.
