# Supplementary material for: Identification of Vesicle‐Mediated Transport‐Related Genes for Predicting Prognosis, Immunotherapy Response, and Drug Screening in Cervical Cancer
Source: Immun Inflamm Dis. 2024 Nov 8;12(11):e70052. doi: 10.1002/iid3.70052 (PMC11544644; doi:10.1002/iid3.70052)
Supplement: Supplementary file 1 — Supplementary Table S1: Vesicle‐mediated transport‐related genes. [file IID3-12-e70052-s003.docx]

| **Supplementary Table 1：Vesicle-mediated transport-related genes** | | |
| --- | --- | --- |
| **Original** | **NCBI (Entrez)** | **Gene** |
| **Member** | **Gene Id** | **Symbol** |
| ENSG00000115977 | [22848](http://view.ncbi.nlm.nih.gov/gene/22848) | [AAK1](http://ensembl.org/Homo_sapiens/Gene/Summary?db=core;g=AAK1) |
| ENSG00000182827 | [64746](http://view.ncbi.nlm.nih.gov/gene/64746) | [ACBD3](http://ensembl.org/Homo_sapiens/Gene/Summary?db=core;g=ACBD3) |
| ENSG00000075624 | [60](http://view.ncbi.nlm.nih.gov/gene/60) | [ACTB](http://ensembl.org/Homo_sapiens/Gene/Summary?db=core;g=ACTB) |
| ENSG00000184009 | [71](http://view.ncbi.nlm.nih.gov/gene/71) | [ACTG1](http://ensembl.org/Homo_sapiens/Gene/Summary?db=core;g=ACTG1) |
| ENSG00000131966 | [55860](http://view.ncbi.nlm.nih.gov/gene/55860) | [ACTR10](http://ensembl.org/Homo_sapiens/Gene/Summary?db=core;g=ACTR10) |
| ENSG00000138107 | [10121](http://view.ncbi.nlm.nih.gov/gene/10121) | [ACTR1A](http://ensembl.org/Homo_sapiens/Gene/Summary?db=core;g=ACTR1A) |
| ENSG00000138071 | [10097](http://view.ncbi.nlm.nih.gov/gene/10097) | [ACTR2](http://ensembl.org/Homo_sapiens/Gene/Summary?db=core;g=ACTR2) |
| ENSG00000115091 | [10096](http://view.ncbi.nlm.nih.gov/gene/10096) | [ACTR3](http://ensembl.org/Homo_sapiens/Gene/Summary?db=core;g=ACTR3) |
| ENSG00000169252 | [154](http://view.ncbi.nlm.nih.gov/gene/154) | [ADRB2](http://ensembl.org/Homo_sapiens/Gene/Summary?db=core;g=ADRB2) |
| ENSG00000173744 | [3267](http://view.ncbi.nlm.nih.gov/gene/3267) | [AGFG1](http://ensembl.org/Homo_sapiens/Gene/Summary?db=core;g=AGFG1) |
| ENSG00000160216 | [56894](http://view.ncbi.nlm.nih.gov/gene/56894) | [AGPAT3](http://ensembl.org/Homo_sapiens/Gene/Summary?db=core;g=AGPAT3) |
| ENSG00000144891 | [185](http://view.ncbi.nlm.nih.gov/gene/185) | [AGTR1](http://ensembl.org/Homo_sapiens/Gene/Summary?db=core;g=AGTR1) |
| ENSG00000142208 | [207](http://view.ncbi.nlm.nih.gov/gene/207) | [AKT1](http://ensembl.org/Homo_sapiens/Gene/Summary?db=core;g=AKT1) |
| ENSG00000105221 | [208](http://view.ncbi.nlm.nih.gov/gene/208) | [AKT2](http://ensembl.org/Homo_sapiens/Gene/Summary?db=core;g=AKT2) |
| ENSG00000117020 | [10000](http://view.ncbi.nlm.nih.gov/gene/10000) | [AKT3](http://ensembl.org/Homo_sapiens/Gene/Summary?db=core;g=AKT3) |
| ENSG00000163631 | 213 | ALB |
| ENSG00000163283 | 250 | ALPP |
| ENSG00000003393 | 57679 | ALS2 |
| ENSG00000178038 | 259173 | ALS2CL |
| ENSG00000106927 | 259 | AMBP |
| ENSG00000078053 | 273 | AMPH |
| ENSG00000029534 | 286 | ANK1 |
| ENSG00000145362 | 287 | ANK2 |
| ENSG00000151150 | 288 | ANK3 |
| ENSG00000105186 | 84079 | ANKRD27 |
| ENSG00000206560 | 23243 | ANKRD28 |
| ENSG00000100280 | 162 | AP1B1 |
| ENSG00000166747 | 164 | AP1G1 |
| ENSG00000213983 | 8906 | AP1G2 |
| ENSG00000072958 | 8907 | AP1M1 |
| ENSG00000129354 | 10053 | AP1M2 |
| ENSG00000106367 | 1174 | AP1S1 |
| ENSG00000182287 | 8905 | AP1S2 |
| ENSG00000152056 | 130340 | AP1S3 |
| ENSG00000196961 | 160 | AP2A1 |
| ENSG00000183020 | 161 | AP2A2 |
| ENSG00000006125 | 163 | AP2B1 |
| ENSG00000161203 | 1173 | AP2M1 |
| ENSG00000042753 | 1175 | AP2S1 |
| ENSG00000132842 | 8546 | AP3B1 |
| ENSG00000177879 | 1176 | AP3S1 |
| ENSG00000134262 | 10717 | AP4B1 |
| ENSG00000081014 | 23431 | AP4E1 |
| ENSG00000221838 | 9179 | AP4M1 |
| ENSG00000100478 | 11154 | AP4S1 |
| ENSG00000118137 | 335 | APOA1 |
| ENSG00000084674 | 338 | APOB |
| ENSG00000130203 | 348 | APOE |
| ENSG00000100342 | 8542 | APOL1 |
| ENSG00000142192 | 351 | APP |
| ENSG00000095139 | 372 | ARCN1 |
| ENSG00000109321 | 374 | AREG |
| ENSG00000143761 | 375 | ARF1 |
| ENSG00000134287 | 377 | ARF3 |
| ENSG00000168374 | 378 | ARF4 |
| ENSG00000004059 | 381 | ARF5 |
| ENSG00000165527 | 382 | ARF6 |
| ENSG00000101199 | 55738 | ARFGAP1 |
| ENSG00000149182 | 84364 | ARFGAP2 |
| ENSG00000242247 | 26286 | ARFGAP3 |
| ENSG00000132254 | 23647 | ARFIP2 |
| ENSG00000101246 | 10139 | ARFRP1 |
| ENSG00000120805 | 400 | ARL1 |
| ENSG00000241685 | 10552 | ARPC1A |
| ENSG00000163466 | 10109 | ARPC2 |
| ENSG00000111229 | 10094 | ARPC3 |
| ENSG00000241553 | 10093 | ARPC4 |
| ENSG00000162704 | 10092 | ARPC5 |
| ENSG00000137486 | 408 | ARRB1 |
| ENSG00000141480 | 409 | ARRB2 |
| ENSG00000169696 | 79058 | ASPSCR1 |
| ENSG00000101200 | 551 | AVP |
| ENSG00000126895 | 554 | AVPR2 |
| ENSG00000105829 | 10282 | BET1 |
| ENSG00000177951 | 51272 | BET1L |
| ENSG00000151746 | 636 | BICD1 |
| ENSG00000185963 | 23299 | BICD2 |
| ENSG00000136717 | 274 | BIN1 |
| ENSG00000135441 | 2647 | BLOC1S1 |
| ENSG00000189114 | 388552 | BLOC1S3 |
| ENSG00000186222 | 55330 | BLOC1S4 |
| ENSG00000104164 | 26258 | BLOC1S6 |
| ENSG00000113734 | 662 | BNIP1 |
| ENSG00000174808 | 685 | BTC |
| ENSG00000111731 | 9847 | C2CD5 |
| ENSG00000198668 | 801 | CALM1 |
| ENSG00000179218 | 811 | CALR |
| ENSG00000116489 | 829 | CAPZA1 |
| ENSG00000198898 | 830 | CAPZA2 |
| ENSG00000177938 | 93661 | CAPZA3 |
| ENSG00000077549 | 832 | CAPZB |
| ENSG00000110395 | 867 | CBL |
| ENSG00000122674 | 51622 | CCZ1 |
| ENSG00000146574 | 221960 | CCZ1B |
| ENSG00000177575 | 9332 | CD163 |
| ENSG00000135218 | 948 | CD36 |
| ENSG00000167286 | 915 | CD3D |
| ENSG00000160654 | 917 | CD3G |
| ENSG00000010610 | 920 | CD4 |
| ENSG00000196352 | 1604 | CD55 |
| ENSG00000085063 | 966 | CD59 |
| ENSG00000138778 | 1062 | CENPE |
| ENSG00000001626 | 1080 | CFTR |
| ENSG00000188419 | 1121 | CHM |
| ENSG00000203668 | 1122 | CHML |
| ENSG00000130724 | 27243 | CHMP2A |
| ENSG00000083937 | 25978 | CHMP2B |
| ENSG00000115561 | 51652 | CHMP3 |
| ENSG00000254505 | 29082 | CHMP4A |
| ENSG00000101421 | 128866 | CHMP4B |
| ENSG00000164695 | 92421 | CHMP4C |
| ENSG00000086065 | 51510 | CHMP5 |
| ENSG00000176108 | 79643 | CHMP6 |
| ENSG00000147457 | 91782 | CHMP7 |
| ENSG00000181072 | 1129 | CHRM2 |
| ENSG00000113282 | 9685 | CLINT1 |
| ENSG00000122705 | 1211 | CLTA |
| ENSG00000175416 | 1212 | CLTB |
| ENSG00000141367 | 1213 | CLTC |
| ENSG00000070371 | 8218 | CLTCL1 |
| ENSG00000177182 | 157807 | CLVS1 |
| ENSG00000146352 | 134829 | CLVS2 |
| ENSG00000100528 | 10175 | CNIH1 |
| ENSG00000174871 | 254263 | CNIH2 |
| ENSG00000143786 | 149111 | CNIH3 |
| ENSG00000166685 | 9382 | COG1 |
| ENSG00000135775 | 22796 | COG2 |
| ENSG00000136152 | 83548 | COG3 |
| ENSG00000103051 | 25839 | COG4 |
| ENSG00000164597 | 10466 | COG5 |
| ENSG00000133103 | 57511 | COG6 |
| ENSG00000168434 | 91949 | COG7 |
| ENSG00000213380 | 84342 | COG8 |
| ENSG00000108821 | 1277 | COL1A1 |
| ENSG00000164692 | 1278 | COL1A2 |
| ENSG00000168542 | 1281 | COL3A1 |
| ENSG00000187498 | 1282 | COL4A1 |
| ENSG00000134871 | 1284 | COL4A2 |
| ENSG00000114270 | 1294 | COL7A1 |
| ENSG00000118004 | 78989 | COLEC11 |
| ENSG00000158270 | 81035 | COLEC12 |
| ENSG00000122218 | 1314 | COPA |
| ENSG00000129083 | 1315 | COPB1 |
| ENSG00000184432 | 9276 | COPB2 |
| ENSG00000105669 | 11316 | COPE |
| ENSG00000181789 | 22820 | COPG1 |
| ENSG00000158623 | 26958 | COPG2 |
| ENSG00000166200 | 9318 | COPS2 |
| ENSG00000141030 | 8533 | COPS3 |
| ENSG00000138663 | 51138 | COPS4 |
| ENSG00000121022 | 10987 | COPS5 |
| ENSG00000168090 | 10980 | COPS6 |
| ENSG00000111652 | 50813 | COPS7A |
| ENSG00000144524 | 64708 | COPS7B |
| ENSG00000198612 | 10920 | COPS8 |
| ENSG00000111481 | 22818 | COPZ1 |
| ENSG00000005243 | 51226 | COPZ2 |
| ENSG00000108582 | 1362 | CPD |
| ENSG00000141551 | 1453 | CSNK1D |
| ENSG00000109861 | 1075 | CTSC |
| ENSG00000101160 | 1522 | CTSZ |
| ENSG00000085733 | 2017 | CTTN |
| ENSG00000257923 | 1523 | CUX1 |
| ENSG00000108669 | 9267 | CYTH1 |
| ENSG00000105443 | 9266 | CYTH2 |
| ENSG00000008256 | 9265 | CYTH3 |
| ENSG00000100055 | 27128 | CYTH4 |
| ENSG00000153071 | 1601 | DAB2 |
| ENSG00000204843 | 1639 | DCTN1 |
| ENSG00000175203 | 10540 | DCTN2 |
| ENSG00000137100 | 11258 | DCTN3 |
| ENSG00000132912 | 51164 | DCTN4 |
| ENSG00000166847 | 84516 | DCTN5 |
| ENSG00000104671 | 10671 | DCTN6 |
| ENSG00000119522 | 57706 | DENND1A |
| ENSG00000213047 | 163486 | DENND1B |
| ENSG00000205744 | 79958 | DENND1C |
| ENSG00000146966 | 27147 | DENND2A |
| ENSG00000166444 | 6764 | DENND2B |
| ENSG00000175984 | 163259 | DENND2C |
| ENSG00000162777 | 79961 | DENND2D |
| ENSG00000105339 | 22898 | DENND3 |
| ENSG00000174485 | 10260 | DENND4A |
| ENSG00000198837 | 9909 | DENND4B |
| ENSG00000137145 | 55667 | DENND4C |
| ENSG00000184014 | 23258 | DENND5A |
| ENSG00000170456 | 160518 | DENND5B |
| ENSG00000174839 | 201627 | DENND6A |
| ENSG00000205593 | 414918 | DENND6B |
| ENSG00000116675 | 9829 | DNAJC6 |
| ENSG00000105612 | 1777 | DNASE2 |
| ENSG00000106976 | 1759 | DNM1 |
| ENSG00000079805 | 1785 | DNM2 |
| ENSG00000197959 | 26052 | DNM3 |
| ENSG00000047579 | 84062 | DTNBP1 |
| ENSG00000004975 | 1856 | DVL2 |
| ENSG00000197102 | 1778 | DYNC1H1 |
| ENSG00000158560 | 1780 | DYNC1I1 |
| ENSG00000077380 | 1781 | DYNC1I2 |
| ENSG00000144635 | 51143 | DYNC1LI1 |
| ENSG00000135720 | 1783 | DYNC1LI2 |
| ENSG00000088986 | 8655 | DYNLL1 |
| ENSG00000264364 | 140735 | DYNLL2 |
| ENSG00000138798 | 1950 | EGF |
| ENSG00000146648 | 1956 | EGFR |
| ENSG00000182585 | 255324 | EPGN |
| ENSG00000063245 | 29924 | EPN1 |
| ENSG00000072134 | 22905 | EPN2 |
| ENSG00000085832 | 2060 | EPS15 |
| ENSG00000127527 | 58513 | EPS15L1 |
| ENSG00000124882 | 2069 | EREG |
| ENSG00000090989 | 55763 | EXOC1 |
| ENSG00000112685 | 55770 | EXOC2 |
| ENSG00000180104 | 11336 | EXOC3 |
| ENSG00000131558 | 60412 | EXOC4 |
| ENSG00000070367 | 10640 | EXOC5 |
| ENSG00000138190 | 54536 | EXOC6 |
| ENSG00000182473 | 23265 | EXOC7 |
| ENSG00000116903 | 149371 | EXOC8 |
| ENSG00000198734 | 2153 | F5 |
| ENSG00000185010 | 2157 | F8 |
| ENSG00000130475 | 23149 | FCHO1 |
| ENSG00000157107 | 115548 | FCHO2 |
| ENSG00000187239 | 23048 | FNBP1 |
| ENSG00000137942 | 54874 | FNBP1L |
| ENSG00000110195 | 2348 | FOLR1 |
| ENSG00000167996 | 2495 | FTH1 |
| ENSG00000087086 | 2512 | FTL |
| ENSG00000174804 | 8322 | FZD4 |
| ENSG00000170296 | 11337 | GABARAP |
| ENSG00000034713 | 11345 | GABARAPL2 |
| ENSG00000178950 | 2580 | GAK |
| ENSG00000141429 | 2589 | GALNT1 |
| ENSG00000143641 | 2590 | GALNT2 |
| ENSG00000165219 | 26130 | GAPVD1 |
| ENSG00000107862 | 8729 | GBF1 |
| ENSG00000179562 | 79571 | GCC1 |
| ENSG00000135968 | 9648 | GCC2 |
| ENSG00000203879 | 2664 | GDI1 |
| ENSG00000057608 | 2665 | GDI2 |
| ENSG00000100083 | 26088 | GGA1 |
| ENSG00000103365 | 23062 | GGA2 |
| ENSG00000125447 | 23163 | GGA3 |
| ENSG00000152661 | 2697 | GJA1 |
| ENSG00000135355 | 84694 | GJA10 |
| ENSG00000121743 | 2700 | GJA3 |
| ENSG00000187513 | 2701 | GJA4 |
| ENSG00000265107 | 2702 | GJA5 |
| ENSG00000121634 | 2703 | GJA8 |
| ENSG00000131233 | 81025 | GJA9 |
| ENSG00000169562 | 2705 | GJB1 |
| ENSG00000165474 | 2706 | GJB2 |
| ENSG00000188910 | 2707 | GJB3 |
| ENSG00000189433 | 127534 | GJB4 |
| ENSG00000189280 | 2709 | GJB5 |
| ENSG00000121742 | 10804 | GJB6 |
| ENSG00000164411 | 375519 | GJB7 |
| ENSG00000182963 | 10052 | GJC1 |
| ENSG00000198835 | 57165 | GJC2 |
| ENSG00000159248 | 57369 | GJD2 |
| ENSG00000183153 | 125111 | GJD3 |
| ENSG00000177291 | 219770 | GJD4 |
| ENSG00000135677 | 2799 | GNS |
| ENSG00000136935 | 2800 | GOLGA1 |
| ENSG00000167110 | 2801 | GOLGA2 |
| ENSG00000144674 | 2803 | GOLGA4 |
| ENSG00000066455 | 9950 | GOLGA5 |
| ENSG00000173230 | 2804 | GOLGB1 |
| ENSG00000173905 | 27333 | GOLIM4 |
| ENSG00000114745 | 64689 | GORASP1 |
| ENSG00000108587 | 9527 | GOSR1 |
| ENSG00000108433 | 9570 | GOSR2 |
| ENSG00000169727 | 2873 | GPS1 |
| ENSG00000177885 | 2885 | GRB2 |
| ENSG00000155511 | 2890 | GRIA1 |
| ENSG00000173020 | 156 | GRK2 |
| ENSG00000100077 | 157 | GRK3 |
| ENSG00000206172 | 3039 | HBA1 |
| ENSG00000188536 | 3040 | HBA2 |
| ENSG00000244734 | 3043 | HBB |
| ENSG00000113070 | 1839 | HBEGF |
| ENSG00000185359 | 9146 | HGS |
| ENSG00000127946 | 3092 | HIP1 |
| ENSG00000130787 | 9026 | HIP1R |
| ENSG00000257017 | 3240 | HP |
| ENSG00000261701 | 3250 | HPR |
| ENSG00000107521 | 3257 | HPS1 |
| ENSG00000100099 | 89781 | HPS4 |
| ENSG00000110169 | 3263 | HPX |
| ENSG00000080824 | 3320 | HSP90AA1 |
| ENSG00000166598 | 7184 | HSP90B1 |
| ENSG00000109971 | 3312 | HSPA8 |
| ENSG00000120694 | 10808 | HSPH1 |
| ENSG00000149428 | 10525 | HYOU1 |
| ENSG00000197081 | 3482 | IGF2R |
| ENSG00000211895 | 3493 | IGHA1 |
| ENSG00000211890 | 3494 | IGHA2 |
| ENSG00000211934 | 28474 | IGHV1-2 |
| ENSG00000211962 | 28465 | IGHV1-46 |
| ENSG00000211973 | 28461 | IGHV1-69 |
| ENSG00000211937 | 28457 | IGHV2-5 |
| ENSG00000274576 | 28454 | IGHV2-70 |
| ENSG00000211941 | 28450 | IGHV3-11 |
| ENSG00000211942 | 28449 | IGHV3-13 |
| ENSG00000211949 | 28442 | IGHV3-23 |
| ENSG00000270550 | 28439 | IGHV3-30 |
| ENSG00000211955 | 28434 | IGHV3-33 |
| ENSG00000211964 | 28424 | IGHV3-48 |
| ENSG00000211967 | 28420 | IGHV3-53 |
| ENSG00000211938 | 28452 | IGHV3-7 |
| ENSG00000211956 | 28395 | IGHV4-34 |
| ENSG00000211959 | 28394 | IGHV4-39 |
| ENSG00000224373 | 28392 | IGHV4-59 |
| ENSG00000243290 | 28940 | IGKV1-12 |
| ENSG00000240864 | 28938 | IGKV1-16 |
| ENSG00000240382 | 28937 | IGKV1-17 |
| ENSG00000242076 | 28933 | IGKV1-33 |
| ENSG00000242371 | 28930 | IGKV1-39 |
| ENSG00000243466 | 28299 | IGKV1-5 |
| ENSG00000278857 | 28903 | IGKV1D-12 |
| ENSG00000241244 | 28901 | IGKV1D-16 |
| ENSG00000239975 | 28896 | IGKV1D-33 |
| ENSG00000251546 | 28893 | IGKV1D-39 |
| ENSG00000244116 | 28921 | IGKV2-28 |
| ENSG00000243238 | 28919 | IGKV2-30 |
| ENSG00000242534 | 28883 | IGKV2D-28 |
| ENSG00000239571 | 28881 | IGKV2D-30 |
| ENSG00000251039 | 28878 | IGKV2D-40 |
| ENSG00000241351 | 28914 | IGKV3-11 |
| ENSG00000244437 | 28913 | IGKV3-15 |
| ENSG00000239951 | 28912 | IGKV3-20 |
| ENSG00000211625 | 28874 | IGKV3D-20 |
| ENSG00000211598 | 28908 | IGKV4-1 |
| ENSG00000211599 | 28907 | IGKV5-2 |
| ENSG00000211677 | 3538 | IGLC2 |
| ENSG00000211679 | 3539 | IGLC3 |
| ENSG00000211653 | 28825 | IGLV1-40 |
| ENSG00000211651 | 28823 | IGLV1-44 |
| ENSG00000211648 | 28822 | IGLV1-47 |
| ENSG00000211644 | 28820 | IGLV1-51 |
| ENSG00000211668 | 28816 | IGLV2-11 |
| ENSG00000211666 | 28815 | IGLV2-14 |
| ENSG00000211660 | 28813 | IGLV2-23 |
| ENSG00000278196 | 28817 | IGLV2-8 |
| ENSG00000211673 | 28809 | IGLV3-1 |
| ENSG00000211663 | 28797 | IGLV3-19 |
| ENSG00000211662 | 28796 | IGLV3-21 |
| ENSG00000211659 | 28793 | IGLV3-25 |
| ENSG00000211658 | 28791 | IGLV3-27 |
| ENSG00000211640 | 28778 | IGLV6-57 |
| ENSG00000211652 | 28776 | IGLV7-43 |
| ENSG00000168685 | 3575 | IL7R |
| ENSG00000254647 | 3630 | INS |
| ENSG00000205726 | 6453 | ITSN1 |
| ENSG00000198399 | 50618 | ITSN2 |
| ENSG00000132465 | 3512 | JCHAIN |
| ENSG00000105438 | 10945 | KDELR1 |
| ENSG00000136240 | 11014 | KDELR2 |
| ENSG00000100196 | 11015 | KDELR3 |
| ENSG00000137261 | 9856 | KIAA0319 |
| ENSG00000138160 | 3832 | KIF11 |
| ENSG00000136883 | 113220 | KIF12 |
| ENSG00000197892 | 23303 | KIF13B |
| ENSG00000163808 | 56992 | KIF15 |
| ENSG00000089177 | 55614 | KIF16B |
| ENSG00000121621 | 81930 | KIF18A |
| ENSG00000186185 | 146909 | KIF18B |
| ENSG00000196169 | 124602 | KIF19 |
| ENSG00000130294 | 547 | KIF1A |
| ENSG00000054523 | 23095 | KIF1B |
| ENSG00000129250 | 10749 | KIF1C |
| ENSG00000112984 | 10112 | KIF20A |
| ENSG00000138182 | 9585 | KIF20B |
| ENSG00000139116 | 55605 | KIF21A |
| ENSG00000116852 | 23046 | KIF21B |
| ENSG00000079616 | 3835 | KIF22 |
| ENSG00000137807 | 9493 | KIF23 |
| ENSG00000125337 | 3834 | KIF25 |
| ENSG00000066735 | 26153 | KIF26A |
| ENSG00000162849 | 55083 | KIF26B |
| ENSG00000165115 | 55582 | KIF27 |
| ENSG00000068796 | 3796 | KIF2A |
| ENSG00000141200 | 84643 | KIF2B |
| ENSG00000142945 | 11004 | KIF2C |
| ENSG00000131437 | 11127 | KIF3A |
| ENSG00000101350 | 9371 | KIF3B |
| ENSG00000084731 | 3797 | KIF3C |
| ENSG00000090889 | 24137 | KIF4A |
| ENSG00000226650 | 285643 | KIF4B |
| ENSG00000155980 | 3798 | KIF5A |
| ENSG00000170759 | 3799 | KIF5B |
| ENSG00000164627 | 221458 | KIF6 |
| ENSG00000088727 | 64147 | KIF9 |
| ENSG00000075945 | 22920 | KIFAP3 |
| ENSG00000204197 | 3833 | KIFC1 |
| ENSG00000167702 | 90990 | KIFC2 |
| ENSG00000126214 | 3831 | KLC1 |
| ENSG00000174996 | 64837 | KLC2 |
| ENSG00000104892 | 147700 | KLC3 |
| ENSG00000137171 | 89953 | KLC4 |
| ENSG00000130164 | 3949 | LDLR |
| ENSG00000157978 | 26119 | LDLRAP1 |
| ENSG00000074695 | 3998 | LMAN1 |
| ENSG00000140506 | 79748 | LMAN1L |
| ENSG00000169223 | 10960 | LMAN2 |
| ENSG00000114988 | 81562 | LMAN2L |
| ENSG00000113441 | 4012 | LNPEP |
| ENSG00000123384 | 4035 | LRP1 |
| ENSG00000081479 | 4036 | LRP2 |
| ENSG00000003056 | 4074 | M6PR |
| ENSG00000110514 | 8567 | MADD |
| ENSG00000111885 | 4121 | MAN1A1 |
| ENSG00000198162 | 10905 | MAN1A2 |
| ENSG00000117643 | 57134 | MAN1C1 |
| ENSG00000112893 | 4124 | MAN2A1 |
| ENSG00000196547 | 4122 | MAN2A2 |
| ENSG00000140941 | 81631 | MAP1LC3B |
| ENSG00000019169 | 8685 | MARCO |
| ENSG00000127241 | 5648 | MASP1 |
| ENSG00000180398 | 90411 | MCFD2 |
| ENSG00000150527 | 4253 | MIA2 |
| ENSG00000154305 | 375056 | MIA3 |
| ENSG00000164077 | 84315 | MON1A |
| ENSG00000103111 | 22879 | MON1B |
| ENSG00000038945 | 4481 | MSR1 |
| ENSG00000141971 | 93343 | MVB12A |
| ENSG00000196814 | 89853 | MVB12B |
| ENSG00000100345 | 4627 | MYH9 |
| ENSG00000197879 | 4641 | MYO1C |
| ENSG00000197535 | 4644 | MYO5A |
| ENSG00000196586 | 4646 | MYO6 |
| ENSG00000139977 | 122830 | NAA30 |
| ENSG00000135040 | 60560 | NAA35 |
| ENSG00000183011 | 84316 | NAA38 |
| ENSG00000105402 | 8775 | NAPA |
| ENSG00000125814 | 63908 | NAPB |
| ENSG00000134265 | 8774 | NAPG |
| ENSG00000151779 | 51594 | NBAS |
| ENSG00000089818 | 25977 | NECAP1 |
| ENSG00000157191 | 55707 | NECAP2 |
| ENSG00000129559 | 4738 | NEDD8 |
| ENSG00000073969 | 4905 | NSF |
| ENSG00000122126 | 4952 | OCRL |
| ENSG00000123240 | 10133 | OPTN |
| ENSG00000124507 | 29993 | PACSIN1 |
| ENSG00000100266 | 11252 | PACSIN2 |
| ENSG00000165912 | 29763 | PACSIN3 |
| ENSG00000007168 | 5048 | PAFAH1B1 |
| ENSG00000168092 | 5049 | PAFAH1B2 |
| ENSG00000079462 | 5050 | PAFAH1B3 |
| ENSG00000073921 | 8301 | PICALM |
| ENSG00000011405 | 5286 | PIK3C2A |
| ENSG00000186111 | 23396 | PIP5K1C |
| ENSG00000116711 | 5321 | PLA2G4A |
| ENSG00000184381 | 8398 | PLA2G6 |
| ENSG00000105355 | 10226 | PLIN3 |
| ENSG00000119414 | 5537 | PPP6C |
| ENSG00000105063 | 22870 | PPP6R1 |
| ENSG00000110075 | 55291 | PPP6R3 |
| ENSG00000138073 | 10113 | PREB |
| ENSG00000162409 | 5563 | PRKAA2 |
| ENSG00000111725 | 5564 | PRKAB1 |
| ENSG00000131791 | 5565 | PRKAB2 |
| ENSG00000181929 | 5571 | PRKAG1 |
| ENSG00000106617 | 51422 | PRKAG2 |
| ENSG00000115592 | 53632 | PRKAG3 |
| ENSG00000134644 | 9698 | PUM1 |
| ENSG00000084733 | 10890 | RAB10 |
| ENSG00000103769 | 8766 | RAB11A |
| ENSG00000185236 | 9230 | RAB11B |
| ENSG00000206418 | 201475 | RAB12 |
| ENSG00000143545 | 5872 | RAB13 |
| ENSG00000119396 | 51552 | RAB14 |
| ENSG00000099246 | 22931 | RAB18 |
| ENSG00000138069 | 5861 | RAB1A |
| ENSG00000174903 | 81876 | RAB1B |
| ENSG00000080371 | 23011 | RAB21 |
| ENSG00000069974 | 5873 | RAB27A |
| ENSG00000041353 | 5874 | RAB27B |
| ENSG00000137502 | 27314 | RAB30 |
| ENSG00000168461 | 11031 | RAB31 |
| ENSG00000118508 | 10981 | RAB32 |
| ENSG00000134594 | 9363 | RAB33A |
| ENSG00000172007 | 83452 | RAB33B |
| ENSG00000111737 | 11021 | RAB35 |
| ENSG00000100228 | 9609 | RAB36 |
| ENSG00000123892 | 23682 | RAB38 |
| ENSG00000179331 | 54734 | RAB39A |
| ENSG00000155961 | 116442 | RAB39B |
| ENSG00000105649 | 5864 | RAB3A |
| ENSG00000115839 | 22930 | RAB3GAP1 |
| ENSG00000118873 | 25782 | RAB3GAP2 |
| ENSG00000167994 | 5866 | RAB3IL1 |
| ENSG00000127328 | 117177 | RAB3IP |
| ENSG00000147127 | 347517 | RAB41 |
| ENSG00000172780 | 339122 | RAB43 |
| ENSG00000168118 | 5867 | RAB4A |
| ENSG00000144566 | 5868 | RAB5A |
| ENSG00000111540 | 5869 | RAB5B |
| ENSG00000108774 | 5878 | RAB5C |
| ENSG00000175582 | 5870 | RAB6A |
| ENSG00000154917 | 51560 | RAB6B |
| ENSG00000075785 | 7879 | RAB7A |
| ENSG00000276600 | 338382 | RAB7B |
| ENSG00000167461 | 4218 | RAB8A |
| ENSG00000166128 | 51762 | RAB8B |
| ENSG00000123595 | 9367 | RAB9A |
| ENSG00000123570 | 51209 | RAB9B |
| ENSG00000029725 | 9135 | RABEP1 |
| ENSG00000136933 | 10244 | RABEPK |
| ENSG00000011454 | 23637 | RABGAP1 |
| ENSG00000154710 | 27342 | RABGEF1 |
| ENSG00000136238 | 5879 | RAC1 |
| ENSG00000161800 | 29127 | RACGAP1 |
| ENSG00000006451 | 5898 | RALA |
| ENSG00000188559 | 57186 | RALGAPA2 |
| ENSG00000170471 | 57148 | RALGAPB |
| ENSG00000135597 | 85021 | REPS1 |
| ENSG00000169891 | 9185 | REPS2 |
| ENSG00000107185 | 9827 | RGP1 |
| ENSG00000164292 | 22836 | RHOBTB3 |
| ENSG00000119729 | 23433 | RHOQ |
| ENSG00000107036 | 57589 | RIC1 |
| ENSG00000174791 | 9610 | RIN1 |
| ENSG00000132669 | 54453 | RIN2 |
| ENSG00000100599 | 79890 | RIN3 |
| ENSG00000187994 | 126432 | RINL |
| ENSG00000135249 | 60561 | RINT1 |
| ENSG00000143947 | 6233 | RPS27A |
| ENSG00000173432 | 6288 | SAA1 |
| ENSG00000152700 | 51128 | SAR1B |
| ENSG00000100241 | 6305 | SBF1 |
| ENSG00000133812 | 81846 | SBF2 |
| ENSG00000168079 | 286133 | SCARA5 |
| ENSG00000073060 | 949 | SCARB1 |
| ENSG00000138760 | 950 | SCARB2 |
| ENSG00000074660 | 8578 | SCARF1 |
| ENSG00000092108 | 23256 | SCFD1 |
| ENSG00000164265 | 117156 | SCGB3A2 |
| ENSG00000153130 | 60592 | SCOC |
| ENSG00000157020 | 6396 | SEC13 |
| ENSG00000148396 | 9919 | SEC16A |
| ENSG00000120341 | 89866 | SEC16B |
| ENSG00000121542 | 26984 | SEC22A |
| ENSG00000265808 | 9554 | SEC22B |
| ENSG00000093183 | 9117 | SEC22C |
| ENSG00000100934 | 10484 | SEC23A |
| ENSG00000107651 | 11196 | SEC23IP |
| ENSG00000113615 | 10802 | SEC24A |
| ENSG00000138802 | 10427 | SEC24B |
| ENSG00000176986 | 9632 | SEC24C |
| ENSG00000150961 | 9871 | SEC24D |
| ENSG00000138674 | 22872 | SEC31A |
| ENSG00000197249 | 5265 | SERPINA1 |
| ENSG00000175793 | 2810 | SFN |
| ENSG00000118473 | 84251 | SGIP1 |
| ENSG00000109686 | 152503 | SH3D19 |
| ENSG00000141985 | 6455 | SH3GL1 |
| ENSG00000107295 | 6456 | SH3GL2 |
| ENSG00000140600 | 6457 | SH3GL3 |
| ENSG00000147010 | 30011 | SH3KBP1 |
| ENSG00000187714 | 6572 | SLC18A3 |
| ENSG00000181856 | 6517 | SLC2A4 |
| ENSG00000136856 | 29988 | SLC2A8 |
| ENSG00000092531 | 8773 | SNAP23 |
| ENSG00000099940 | 9342 | SNAP29 |
| ENSG00000065609 | 9892 | SNAP91 |
| ENSG00000143553 | 23557 | SNAPIN |
| ENSG00000159210 | 11267 | SNF8 |
| ENSG00000178996 | 112574 | SNX18 |
| ENSG00000205302 | 6643 | SNX2 |
| ENSG00000089006 | 27131 | SNX5 |
| ENSG00000130340 | 51429 | SNX9 |
| ENSG00000134243 | 6272 | SORT1 |
| ENSG00000113140 | 6678 | SPARC |
| ENSG00000163554 | 6708 | SPTA1 |
| ENSG00000197694 | 6709 | SPTAN1 |
| ENSG00000070182 | 6710 | SPTB |
| ENSG00000115306 | 6711 | SPTBN1 |
| ENSG00000173898 | 6712 | SPTBN2 |
| ENSG00000160460 | 57731 | SPTBN4 |
| ENSG00000137877 | 51332 | SPTBN5 |
| ENSG00000197122 | 6714 | SRC |
| ENSG00000179954 | 284297 | SSC5D |
| ENSG00000010327 | 23166 | STAB1 |
| ENSG00000136011 | 55576 | STAB2 |
| ENSG00000136738 | 8027 | STAM |
| ENSG00000115145 | 10254 | STAM2 |
| ENSG00000243244 | 11037 | STON1 |
| ENSG00000140022 | 85439 | STON2 |
| ENSG00000104915 | 8677 | STX10 |
| ENSG00000124222 | 8675 | STX16 |
| ENSG00000136874 | 55014 | STX17 |
| ENSG00000168818 | 53407 | STX18 |
| ENSG00000103496 | 6810 | STX4 |
| ENSG00000162236 | 6811 | STX5 |
| ENSG00000135823 | 10228 | STX6 |
| ENSG00000116266 | 6814 | STXBP3 |
| ENSG00000148248 | 6836 | SURF4 |
| ENSG00000159082 | 8867 | SYNJ1 |
| ENSG00000078269 | 8871 | SYNJ2 |
| ENSG00000204070 | 90196 | SYS1 |
| ENSG00000067715 | 6857 | SYT1 |
| ENSG00000132718 | 23208 | SYT11 |
| ENSG00000143858 | 127833 | SYT2 |
| ENSG00000149043 | 90019 | SYT8 |
| ENSG00000170743 | 143425 | SYT9 |
| ENSG00000142765 | 84958 | SYTL1 |
| ENSG00000115353 | 6869 | TACR1 |
| ENSG00000065882 | 23216 | TBC1D1 |
| ENSG00000099992 | 83874 | TBC1D10A |
| ENSG00000169221 | 26000 | TBC1D10B |
| ENSG00000175463 | 374403 | TBC1D10C |
| ENSG00000107021 | 54662 | TBC1D13 |
| ENSG00000132405 | 57533 | TBC1D14 |
| ENSG00000121749 | 64786 | TBC1D15 |
| ENSG00000167291 | 125058 | TBC1D16 |
| ENSG00000104946 | 79735 | TBC1D17 |
| ENSG00000095383 | 55357 | TBC1D2 |
| ENSG00000125875 | 128637 | TBC1D20 |
| ENSG00000162065 | 57465 | TBC1D24 |
| ENSG00000068354 | 4943 | TBC1D25 |
| ENSG00000274611 | 729873 | TBC1D3 |
| ENSG00000136111 | 9882 | TBC1D4 |
| ENSG00000145979 | 51256 | TBC1D7 |
| ENSG00000133138 | 54885 | TBC1D8B |
| ENSG00000091513 | 7018 | TF |
| ENSG00000114354 | 10342 | TFG |
| ENSG00000072274 | 7037 | TFRC |
| ENSG00000163235 | 7039 | TGFA |
| ENSG00000152291 | 10618 | TGOLN2 |
| ENSG00000104067 | 7082 | TJP1 |
| ENSG00000170348 | 10972 | TMED10 |
| ENSG00000086598 | 10959 | TMED2 |
| ENSG00000166557 | 23423 | TMED3 |
| ENSG00000134970 | 51014 | TMED7 |
| ENSG00000184840 | 54732 | TMED9 |
| ENSG00000126062 | 11070 | TMEM115 |
| ENSG00000144747 | 7110 | TMF1 |
| ENSG00000136827 | 1861 | TOR1A |
| ENSG00000136816 | 27348 | TOR1B |
| ENSG00000076554 | 7163 | TPD52 |
| ENSG00000111907 | 7164 | TPD52L1 |
| ENSG00000170043 | 58485 | TRAPPC1 |
| ENSG00000160218 | 7109 | TRAPPC10 |
| ENSG00000168538 | 60684 | TRAPPC11 |
| ENSG00000171853 | 51112 | TRAPPC12 |
| ENSG00000113597 | 80006 | TRAPPC13 |
| ENSG00000196459 | 6399 | TRAPPC2 |
| ENSG00000167515 | 51693 | TRAPPC2L |
| ENSG00000054116 | 27095 | TRAPPC3 |
| ENSG00000196655 | 51399 | TRAPPC4 |
| ENSG00000181029 | 126003 | TRAPPC5 |
| ENSG00000007255 | 79090 | TRAPPC6A |
| ENSG00000182400 | 122553 | TRAPPC6B |
| ENSG00000153339 | 22878 | TRAPPC8 |
| ENSG00000167632 | 83696 | TRAPPC9 |
| ENSG00000125733 | 9322 | TRIP10 |
| ENSG00000100815 | 9321 | TRIP11 |
| ENSG00000165699 | 7248 | TSC1 |
| ENSG00000103197 | 7249 | TSC2 |
| ENSG00000074319 | 7251 | TSG101 |
| ENSG00000167552 | 7846 | TUBA1A |
| ENSG00000123416 | 10376 | TUBA1B |
| ENSG00000167553 | 84790 | TUBA1C |
| ENSG00000198033 | 7278 | TUBA3C |
| ENSG00000075886 | 113457 | TUBA3D |
| ENSG00000152086 | 112714 | TUBA3E |
| ENSG00000127824 | 7277 | TUBA4A |
| ENSG00000243910 | 80086 | TUBA4B |
| ENSG00000183785 | 51807 | TUBA8 |
| ENSG00000178462 | 79861 | TUBAL3 |
| ENSG00000101162 | 81027 | TUBB1 |
| ENSG00000137267 | 7280 | TUBB2A |
| ENSG00000137285 | 347733 | TUBB2B |
| ENSG00000258947 | 10381 | TUBB3 |
| ENSG00000104833 | 10382 | TUBB4A |
| ENSG00000188229 | 10383 | TUBB4B |
| ENSG00000176014 | 84617 | TUBB6 |
| ENSG00000261456 | 347688 | TUBB8 |
| ENSG00000173213 | 260334 | TUBB8B |
| ENSG00000239264 | 81567 | TXNDC5 |
| ENSG00000221983 | 7311 | UBA52 |
| ENSG00000165006 | 51271 | UBAP1 |
| ENSG00000170315 | 7314 | UBB |
| ENSG00000150991 | 7316 | UBC |
| ENSG00000135018 | 29979 | UBQLN1 |
| ENSG00000188021 | 29978 | UBQLN2 |
| ENSG00000177169 | 8408 | ULK1 |
| ENSG00000053501 | 55850 | USE1 |
| ENSG00000138768 | 8615 | USO1 |
| ENSG00000148429 | 9712 | USP6NL |
| ENSG00000220205 | 6844 | VAMP2 |
| ENSG00000049245 | 9341 | VAMP3 |
| ENSG00000117533 | 8674 | VAMP4 |
| ENSG00000124333 | 6845 | VAMP7 |
| ENSG00000118640 | 8673 | VAMP8 |
| ENSG00000131475 | 84313 | VPS25 |
| ENSG00000160948 | 51160 | VPS28 |
| ENSG00000136100 | 51028 | VPS36 |
| ENSG00000155975 | 137492 | VPS37A |
| ENSG00000139722 | 79720 | VPS37B |
| ENSG00000167987 | 55048 | VPS37C |
| ENSG00000176428 | 155382 | VPS37D |
| ENSG00000136631 | 11311 | VPS45 |
| ENSG00000132612 | 27183 | VPS4A |
| ENSG00000119541 | 9525 | VPS4B |
| ENSG00000149823 | 738 | VPS51 |
| ENSG00000206286 | 6293 | VPS52 |
| ENSG00000141252 | 55275 | VPS53 |
| ENSG00000143952 | 51542 | VPS54 |
| ENSG00000009844 | 51534 | VTA1 |
| ENSG00000151532 | 143187 | VTI1A |
| ENSG00000106299 | 8976 | WASL |
| ENSG00000114251 | 7474 | WNT5A |
| ENSG00000181704 | 286451 | YIPF6 |
| ENSG00000106636 | 10652 | YKT6 |
| ENSG00000166913 | 7529 | YWHAB |
| ENSG00000108953 | 7531 | YWHAE |
| ENSG00000170027 | 7532 | YWHAG |
| ENSG00000128245 | 7533 | YWHAH |
| ENSG00000134308 | 10971 | YWHAQ |
| ENSG00000164924 | 7534 | YWHAZ |
| ENSG00000086827 | 9183 | ZW10 |
