# Supplementary material for: Identification of Vesicle‐Mediated Transport‐Related Genes for Predicting Prognosis, Immunotherapy Response, and Drug Screening in Cervical Cancer
Source: Immun Inflamm Dis. 2024 Nov 8;12(11):e70052. doi: 10.1002/iid3.70052 (PMC11544644; doi:10.1002/iid3.70052)
Supplement: Supplementary file 2 — Supplementary Table S2: 354 intersecting genes. [file IID3-12-e70052-s001.docx]

| Supplementary Table S2：354 intersect genes(DE-VMTRGs) |
| --- |
| DVL2 |
| COPZ2 |
| RALA |
| PAFAH1B1 |
| TRAPPC6A |
| CYTH3 |
| STAB1 |
| CD4 |
| MARCO |
| GABARAPL2 |
| RAB27B |
| AP2S1 |
| GDI2 |
| EPN1 |
| TBC1D1 |
| GOLGA5 |
| KIF2A |
| CLTCL1 |
| EPN2 |
| TFRC |
| AP1M1 |
| PICALM |
| NSF |
| SCARF1 |
| LMAN1 |
| ACTB |
| RAB7A |
| TPD52 |
| DYNC1I2 |
| SYNJ2 |
| PAFAH1B3 |
| KIF22 |
| DNM2 |
| RAB21 |
| HSP90AA1 |
| RAB10 |
| CD59 |
| CTTN |
| CHMP5 |
| TMED2 |
| ZW10 |
| FTL |
| KIF9 |
| SNX5 |
| KIF16B |
| KIF4A |
| SCFD1 |
| SNAP23 |
| SEC22C |
| TBC1D2 |
| SNAP29 |
| TBC1D10A |
| GGA1 |
| KDELR3 |
| AP1B1 |
| APOL1 |
| SEC23A |
| CTSZ |
| ARFGAP1 |
| ARFRP1 |
| KIF3B |
| CHMP4B |
| COG4 |
| TSC2 |
| STX4 |
| KLC3 |
| TBC1D17 |
| PPP6R1 |
| AKT2 |
| DENND3 |
| PLIN3 |
| NAPA |
| KDELR1 |
| CYTH2 |
| DNASE2 |
| AP1S1 |
| PRKAG2 |
| YKT6 |
| DNM1 |
| RGP1 |
| GOSR2 |
| CPD |
| COL1A1 |
| AREG |
| SH3D19 |
| HSPA8 |
| MADD |
| MAN1A1 |
| EXOC2 |
| KIF20A |
| HBEGF |
| SPARC |
| WNT5A |
| GORASP1 |
| SPTBN1 |
| CHMP3 |
| CAPZA1 |
| EXOC8 |
| AKT3 |
| MAN1C1 |
| RAB32 |
| VAMP8 |
| RAB14 |
| PPP6C |
| VPS4B |
| RHOQ |
| HSPH1 |
| KIF18A |
| GJB6 |
| TBC1D15 |
| COPA |
| CCZ1 |
| LRP1 |
| RAB38 |
| VAMP7 |
| TRIP10 |
| KLC1 |
| MASP1 |
| EPS15L1 |
| TUBA4A |
| YWHAH |
| AP1M2 |
| NEDD8 |
| SNX9 |
| FCHO1 |
| VPS25 |
| ACTR10 |
| ARFIP2 |
| TBC1D14 |
| RIN2 |
| SYT11 |
| SBF2 |
| ARF3 |
| YWHAQ |
| COL4A2 |
| TMED7 |
| REPS1 |
| DYNC1LI2 |
| STX6 |
| GCC2 |
| RAC1 |
| KDELR2 |
| BIN1 |
| TOR1B |
| DCTN3 |
| DENND4C |
| KLC4 |
| TUBB2B |
| ARRB1 |
| RAB30 |
| KIF23 |
| SPTBN5 |
| ACTR2 |
| KIF11 |
| KIF20B |
| SEC31A |
| CENPE |
| VPS37B |
| STON2 |
| MAP1LC3B |
| COPS3 |
| GALNT1 |
| CSNK1D |
| MVB12A |
| AKT1 |
| SYTL1 |
| KIF2C |
| SNAPIN |
| GALNT2 |
| RPS27A |
| COPS7B |
| CCZ1B |
| DENND2A |
| SURF4 |
| SYT8 |
| VPS51 |
| MIA2 |
| SEC24D |
| UBC |
| ANK3 |
| BICD1 |
| AP1S3 |
| GJA1 |
| DAB2 |
| RABGEF1 |
| VPS37A |
| LDLRAP1 |
| COLEC12 |
| SNF8 |
| TRAPPC10 |
| CD3G |
| VPS28 |
| RACGAP1 |
| DENND2D |
| KIF26B |
| TGFA |
| KIF15 |
| RHOBTB3 |
| COG5 |
| COL1A2 |
| CHMP4C |
| YWHAZ |
| GJB2 |
| ARF6 |
| PACSIN3 |
| DENND2B |
| TMED3 |
| HSP90B1 |
| COG1 |
| YWHAB |
| GOLGA2 |
| CD3D |
| TRAPPC2L |
| TUBA1A |
| TUBA1C |
| RAB3IL1 |
| FTH1 |
| COL3A1 |
| STX18 |
| LMAN2 |
| ASPSCR1 |
| YWHAG |
| TRAPPC1 |
| GABARAP |
| DENND5B |
| KIF5B |
| TRAPPC12 |
| SAA1 |
| SPTBN2 |
| GOLIM4 |
| FZD4 |
| CNIH2 |
| RAB1B |
| DCTN2 |
| CLTB |
| SFN |
| VPS37D |
| SEC24C |
| ULK1 |
| CD163 |
| GRB2 |
| GAK |
| CALR |
| GCC1 |
| SSC5D |
| EXOC3 |
| AP1S2 |
| EXOC7 |
| ACBD3 |
| GJC1 |
| AP2A2 |
| ACTG1 |
| DENND5A |
| PLA2G6 |
| TMED9 |
| F8 |
| HGS |
| BICD2 |
| KIF18B |
| BLOC1S4 |
| FNBP1 |
| GJA4 |
| RINL |
| UBQLN2 |
| TUBB4B |
| HBA2 |
| GJB3 |
| BLOC1S3 |
| GJB5 |
| GJB4 |
| MAN2A2 |
| MYO6 |
| TRAPPC4 |
| MVB12B |
| AP2A1 |
| DYNC1H1 |
| SERPINA1 |
| SPTAN1 |
| MYO1C |
| ITSN2 |
| COPS8 |
| GJC2 |
| DENND4B |
| CAPZA2 |
| CHML |
| GDI1 |
| KIFC1 |
| DCTN1 |
| ITSN1 |
| DENND1C |
| VPS52 |
| ANKRD28 |
| IGKV4-1 |
| IGKV5-2 |
| IGKV3D-20 |
| IGLV6-57 |
| IGLV1-51 |
| IGLV1-47 |
| IGLV1-44 |
| IGLV7-43 |
| IGLV1-40 |
| IGLV3-27 |
| IGLV3-25 |
| IGLV2-23 |
| IGLV3-21 |
| IGLV3-19 |
| IGLV2-14 |
| IGLV2-11 |
| IGLV3-1 |
| IGLC2 |
| IGLC3 |
| IGHA2 |
| IGHA1 |
| IGHV1-2 |
| IGHV3-11 |
| IGHV3-13 |
| IGHV3-23 |
| IGHV3-33 |
| IGHV4-34 |
| IGHV4-39 |
| IGHV1-46 |
| IGHV3-48 |
| IGHV3-53 |
| IGHV1-69 |
| DENND1B |
| COG8 |
| AP1G2 |
| VAMP2 |
| UBA52 |
| IGHV4-59 |
| TXNDC5 |
| IGKV3-20 |
| IGKV1-17 |
| IGKV1-16 |
| IGKV1D-16 |
| IGKV3-11 |
| ARPC1A |
| IGKV1-33 |
| STON1 |
| IGKV1-5 |
| IGKV2-28 |
| IGKV3-15 |
| HBB |
| IGKV1D-39 |
| CHMP4A |
| HP |
| CUX1 |
| DYNLL2 |
| GJA5 |
| SEC22B |
| IGHV3-30 |
| IGHV2-70 |
| RAB7B |
| IGLV2-8 |
