# Supplementary material for: Identification of Vesicle‐Mediated Transport‐Related Genes for Predicting Prognosis, Immunotherapy Response, and Drug Screening in Cervical Cancer
Source: Immun Inflamm Dis. 2024 Nov 8;12(11):e70052. doi: 10.1002/iid3.70052 (PMC11544644; doi:10.1002/iid3.70052)
Supplement: Supplementary file 3 — Supplementary Table S3: Results of Univariate Cox analysis. [file IID3-12-e70052-s002.docx]

| Supplementary Table S3: Results of Univariate Cox analysis | | | | | |
| --- | --- | --- | --- | --- | --- |
| gene | HR | HR.95L | HR.95H | z | pvalue |
| HGS | 2.843601297 | 1.624002787 | 4.979097575 | 3.656524347 | 0.000255658 |
| CAPZA2 | 2.235967315 | 1.247953895 | 4.006197546 | 2.704418425 | 0.006842407 |
| CHMP4C | 1.910866816 | 1.240540077 | 2.943405099 | 2.937867017 | 0.003304787 |
| KIF26B | 1.626979748 | 1.162737131 | 2.276579141 | 2.839613956 | 0.004516816 |
| CD3D | 0.769720131 | 0.646606324 | 0.916274799 | -2.943255423 | 0.003247803 |
| TXNDC5 | 1.844120485 | 1.201052826 | 2.831499408 | 2.797322239 | 0.005152811 |
| COPA | 2.132428188 | 1.261073699 | 3.605855854 | 2.825454157 | 0.004721364 |
| DENND2D | 0.517359481 | 0.327532732 | 0.81720331 | -2.825440965 | 0.004721559 |
| YKT6 | 2.451325502 | 1.405528131 | 4.275258947 | 3.159493912 | 0.001580434 |
| SPTBN1 | 1.812502229 | 1.200610665 | 2.736244501 | 2.829980866 | 0.004655079 |
| DNASE2 | 0.568672523 | 0.371200516 | 0.871196091 | -2.593531145 | 0.009499593 |
| ACBD3 | 1.999527927 | 1.21663423 | 3.286207008 | 2.733531078 | 0.006265921 |
| TFRC | 1.374263586 | 1.138409018 | 1.658982295 | 3.309363326 | 0.000935084 |
| CD3G | 0.600063232 | 0.41181457 | 0.874364115 | -2.658950046 | 0.007838458 |
| KDELR2 | 1.827547033 | 1.19048772 | 2.80551248 | 2.757295089 | 0.005828173 |
| SEC31A | 1.947586692 | 1.19899499 | 3.163561111 | 2.69320692 | 0.007076833 |
| DENND4C | 1.92693621 | 1.205115988 | 3.08110024 | 2.739078978 | 0.006161157 |
| MAN1C1 | 0.516845526 | 0.316039675 | 0.845239755 | -2.629926078 | 0.008540344 |
| SEC23A | 1.840511685 | 1.247977838 | 2.71437774 | 3.077489616 | 0.002087521 |
| YWHAG | 2.131796623 | 1.410757132 | 3.221360176 | 3.593715408 | 0.000325996 |
| TGFA | 1.462420454 | 1.152281437 | 1.856034051 | 3.12553517 | 0.001774818 |
| SBF2 | 2.240434147 | 1.236553712 | 4.059302173 | 2.660160373 | 0.007810345 |
| RGP1 | 1.878222776 | 1.167483524 | 3.021645037 | 2.598275862 | 0.009369319 |
| KIF22 | 0.496040316 | 0.32219328 | 0.763690649 | -3.184494015 | 0.001450073 |
| TMED2 | 1.993392093 | 1.201640111 | 3.306823733 | 2.671255873 | 0.007556802 |
