# Supplementary material for: Identification of Vesicle‐Mediated Transport‐Related Genes for Predicting Prognosis, Immunotherapy Response, and Drug Screening in Cervical Cancer
Source: Immun Inflamm Dis. 2024 Nov 8;12(11):e70052. doi: 10.1002/iid3.70052 (PMC11544644; doi:10.1002/iid3.70052)
Supplement: Supplementary file 4 — Supplementary Table S4: Results of LASSO regression analysis. lasso, least absolute shrinkage and selection operator. [file IID3-12-e70052-s004.docx]

| Supplementary Table S4：Results of LASSO regression analysis. lasso, least absolute shrinkage and selection operator | |
| --- | --- |
| Gene | Coef |
| HGS | 0.67030147 |
| CAPZA2 | 0.599555231 |
| CHMP4C | 0.368756377 |
| KIF26B | 0.267278277 |
| DENND2D | -0.300214617 |
| YKT6 | 0.065489752 |
| DNASE2 | -0.121918277 |
| TFRC | 0.188994452 |
| KDELR2 | 0.503595975 |
| SEC31A | 0.289627017 |
| MAN1C1 | -0.383501655 |
| SEC23A | 0.07803807 |
| TGFA | 0.119732998 |
| KIF22 | -0.041852258 |
